# Institutional trust and vaccination delay as key metrics for vaccination rollout success

**DOI:** 10.1038/s43856-026-01597-4

**Published:** 2026-04-29

**Authors:** Cyrus Lap Kwan Leung, Kin-Kit Li, Arthur Tang, Wilson Wai San Tam, Samuel Yeung Shan Wong, Wan In Wei, Kin On Kwok

**Affiliations:** 1 JC School of Public Health and Primary Care, The Chinese University of Hong Kong, New Territories, Hong Kong; 2 Department of Social and Behavioural Sciences, City University of Hong Kong, Kowloon, Hong Kong; 3 School of Science, Engineering and Technology, RMIT University, Ho Chi Minh City, Vietnam; 4 Alice Lee Centre for Nursing Studies, Yong Loo Lin School of Medicine, National University of Singapore, Singapore, Singapore; 5 Hong Kong Institute of Asia-Pacific Studies, The Chinese University of Hong Kong, New Territories, Hong Kong; 6 Department of Infectious Disease Epidemiology, School of Public Health, Imperial College London, London, UK

**Keywords:** Preventive medicine, Infectious diseases, Epidemiology

## Abstract

**Background:**

Timely vaccination effectively reduced COVID-19 hospitalizations and mortality, yet vaccination hesitancy undermined this benefit. Understanding the factors contributing to hesitancy is critical for improving future pandemic control by identifying barriers to timely vaccination. This paper operationalizes hesitancy in terms of vaccine delay—a key public health metric that reflects changing vaccination policies and infection status, factors that can alter individuals’ eligibility, and real-world complexities like infections.

**Method:**

Using longitudinal data from the earliest stage of the pandemic in Hong Kong, we examined how institutional trust and the 5C constructs (confidence, complacency, constraints, calculation, and collective responsibility) influenced both vaccination intention and timing.

**Results:**

Our results show that only 34.89% and 42.97% of vaccinated participants received their first and third doses within 100 days of eligibility, respectively, despite rising uptake prior to government mandates. Confidence and vaccination intention are key predictors of delay, and higher institutional trust boosts both confidence and collective responsibility, thereby enhancing intention and reducing delays.

**Conclusions:**

These findings underscore the importance of building institutional trust and public confidence to minimize vaccine delay, particularly among vulnerable populations. Ultimately, incorporating vaccine delay as a key metric into public health strategies can guide more effective interventions and strengthen pandemic preparedness.

## Plain language summary

Vaccination against COVID-19 has prevented many hospitalizations and deaths, yet delays in getting vaccinated have limited these benefits. We examined why people in Hong Kong postponed vaccination and proposed vaccination delay as an important metric for understanding vaccine hesitancy and rollout success. Using repeated survey data collected during the pandemic, we analyzed how trust in health authorities and psychological factors—such as confidence, perceived barriers, and social responsibility—affected both vaccination intention and timing. We found that higher trust and confidence were linked to earlier vaccination. Recognizing vaccination delay as a key public health measure can help governments design strategies that build trust, encourage timely uptake, and improve preparedness for future pandemics.

## Abstract (web summary)

Leung et al. examine how institutional trust and psychological factors shape vaccination timing in Hong Kong. Higher trust and confidence lead to earlier vaccination, highlighting vaccination delay as an important metric for evaluating vaccine rollout success

## Introduction

In the face of pandemics, timely and extensive vaccination is a potent public health strategy to mitigate the spread of infectious diseases and reduce severe cases and hospitalizations^1,2^. Despite the rapid rollout of vaccination campaigns during the COVID-19 pandemic, a significant challenge emerged beyond outright vaccine skepticism: Vaccine hesitancy among individuals who did not fundamentally oppose vaccination^3^, often overlooked in the binary discourse of pro- and anti-vaccination attitudes. Individuals such as “vaccine fence-sitters,” who are equivocal in their attitude towards vaccines, and “vaccine apathetics,” who are disengaged from vaccine-related information, represent key targets for public health intervention because of their prevalence and potentially modifiable attitudes^4,5^. Capturing their hesitancy in terms of delay in vaccination, rather than merely measuring intentions or end status, provides a more behaviorally grounded approach to understanding this middle-ground group. Here, we conceptualize vaccine hesitancy not only as an attitude but also as a behavioral manifestation, reflected in the postponement of vaccination despite eventual acceptance.

The failure in public health interventions to address vaccination hesitancy underscores a key divide in perspectives between two significant stakeholders: Experts and healthcare authorities tend to emphasize vaccine confidence and the rarity of adverse events, whereas hesitant individuals often focus on trust, especially trust in vaccine efficacy and safety, in parties manufacturing and administering vaccines, and in the healthcare system more broadly^6^, viewing vaccination as a risk-laden decision^7^. In fact, although trust has long been recognized as a central determinant of vaccine acceptance, its role became particularly visible and widely discussed during the COVID-19 pandemic^8–10^, when confidence in health authorities and vaccine safety came under intense public scrutiny.

To characterize the psychological dimensions through which institutional trust shapes vaccination behavior, we adopt the 5C psychological antecedents of vaccination (confidence, complacency, constraints, calculation, and collective responsibility), a simple and well-validated framework widely applied and validated during the COVID-19 pandemic to understand the psychological factors predicting vaccine hesitancy^11,12^. The 5C model builds on the WHO’s 3C framework by incorporating additional trust-related dimensions such as collective responsibility^11,12^. Well-established models such as the Vaccine Confidence Index^13^ and the Increasing Vaccination Model^14^ inform vaccination behavior, also incorporating elements of trust, but the 5C model offers stronger empirical support, cross-cultural validation, and clear operationalization of trust-related constructs. Features of the vaccination rollout, such as vaccine type and provider^15^, as well as individual medical and demographic factors like a history of COVID-19 infection in oneself or close family members^16^, are likely to shape vaccination behavior through their influence on 5C constructs, such as confidence (e.g., perceived vaccine safety), constraints (e.g., accessibility), complacency (e.g., perceived necessity of vaccination), and collective responsibility (e.g., motivation to protect others).

Institutional trust plays a pivotal role in shaping vaccine hesitancy and each of the 5C constructs by influencing how individuals perceive vaccines, risks, and public health messaging^17,18^. Compared to trust in the vaccine itself, institutional trust, such as trust in the government, health authorities, and medical institutions, broadly informs people’s acceptance of official guidance, credibility of information, and the perceived fairness and efficiency of vaccine delivery^17^. In emotionally charged or uncertain contexts like the pandemic, institutional trust may be a stronger determinant of vaccine uptake than technical confidence in the vaccine alone^19^. Exploring further, confidence, which is about trust in the vaccine’s efficacy and safety and in the healthcare system, is deeply rooted in the public’s perception of the integrity of vaccine research and the healthcare authorities^11,20,21^ and underpins the public’s willingness to accept vaccines. Complacency arises from a low perceived risk of vaccine-preventable diseases. Trustworthy and clear communication can effectively mitigate complacency by accurately conveying the risks associated with vaccine-preventable diseases e.g.,^22–24^. Calculation in the 5C involves the deliberate seeking and evaluation of vaccine-related information. People are more likely to seek additional information from sources they already perceive as credible and trustworthy^25^. Lastly, collective responsibility, which is about societal considerations such as achieving herd immunity and protecting others, can be strengthened by a sense of trust in the community that other members are also doing their job to get vaccinated^26^. Although constraints, which refer to external barriers such as scheduling or logistics, do not directly map onto trust^7^, they may be alleviated when individuals trust that the healthcare system facilitates rather than hinders access. The relationship between institutional trust and these perceived barriers also depends on the broader social, cultural, and political context. Factors such as political rhetoric, media narratives, and structural inequalities can shape whether people view vaccination systems as enabling or obstructive, thereby influencing access and acceptance. Besides the 5C model, trust is a fundamental component in other widely used behavioral change models in vaccination behavior, such as the Health Belief Model^27^ and the Theory of Planned Behavior (TPB)^28^.

In this study, we aim to investigate the factors contributing to vaccination hesitancy, operationalized as the delay in administering vaccines after their availability to the public, using structural equation modeling (SEM) and Cox proportional-hazards regression. SEM allows us to assess the pathways from institutional trust through the 5 C components to intention, while Cox regression evaluates the timing of vaccination in relation to these psychological constructs. This combination offers a comprehensive understanding of both the psychological and temporal predictors of vaccination delay.

Focusing on delay enables a behavioral operationalization of vaccine hesitancy that extends beyond attitudinal measures. The delay measure provides a more accurate reflection of barriers to timely vaccination and the effectiveness of public health interventions, offering deeper insights into the factors influencing delayed decisions among individuals who do not explicitly reject vaccines. We adopt the TPB to frame how institutional trust and the 5C constructs influence vaccination timing. In our model, the 5C constructs align with TPB components: Confidence aligns with attitudes, collective responsibility with perceived norms, and constraints with perceived behavioral control. This conceptual integration improves interpretability and supports an integrated understanding of how upstream trust impacts downstream behavior. The longitudinal design further allows us to examine how these psychological pathways unfold over time in response to the progression of the pandemic and the implementation of public health policies. The study period spanned both a voluntary phase before vaccine mandates were introduced and a later period of phased policy implementation, capturing behavioral variation in vaccination timing that reflects both individual autonomy and responsiveness to evolving public health policies. The results show that higher institutional trust enhances confidence and collective responsibility, increases vaccination intention, and shortens vaccination delay, highlighting the importance of institutional trust and vaccination delay as key metrics for evaluating vaccine rollout success and pandemic preparedness.

## Methods

### Cohort establishment

The community cohort was initiated within 36 h of the first confirmed COVID-19 case in Hong Kong through an open community-based online survey^29^. To achieve broad geographic coverage across all 18 administrative districts, we contacted all 452 elected District Councilors and their chairpersons or vice-chairpersons via email and publicly listed phone numbers. Councilors were invited to disseminate the survey link through their usual communication channels. The survey was open to all adults aged 18 or above who could read Chinese and had lived in Hong Kong for at least 5 days per week over the past month. Participation was voluntary, and each device could only submit the survey once to prevent duplicate entries. Of the individuals who accessed the survey, 1715 provided consent, with 1712 answering the vaccination-related variables. While the sample was not probabilistically drawn, the district-level outreach strategy enhanced regional representativeness. Participation was anonymous, and we did not track the total number of individuals reached by the councilors. Participants recruited in Round 1 were recontacted in subsequent rounds to form a longitudinal panel, and they provided informed consent before completing the surveys of each round. The cohort was topped up in R10 and R11. The covered periods and the sample sizes of each round are presented in Table S1. The number of participants per round was also constrained by the available research funding at that time.

### Study timeline

The cohort study covered different pandemic stages in Hong Kong (Table S1). Our analyses used data from R4 onwards, when the research team first incorporated the 5C constructs of vaccine hesitancy into the survey to examine public attitudes prior to the local availability of COVID-19 vaccines. Participants without data from R8 onwards (when COVID-19 vaccines became available in Hong Kong), without vaccination records, or who claimed an earlier-than-available date of vaccination were excluded (see Fig. S2 and Supplementary Materials).

### Measures

The survey included demographics, health conditions, risk perception, psychosocial health, institutional trust, and perceptions of preventive measures. We used the 5C psychological antecedents of the vaccination^11^ to measure the five domains of vaccine hesitancy, namely, confidence, complacency, constraints, calculation, and collective responsibility. Participants rated their extent of agreeing with the fifteen statements on the scale on a 7-point Likert scale (1 = *strongly disagree*; 7 = *strongly agree*), and higher domain scores indicate stronger agreement of those domains. As in our prior work^4^, the negatively worded item for collective responsibility was excluded due to its poor convergence with the other items in the subscale.

The trust in the government’s response to the COVID-19 pandemic was measured using the 5-item government trust measure^30^. We adapted the statements to fit the context of the COVID-19 pandemic (for example, “How much trust do you have in the government with respect to fighting the COVID-19 pandemic?”). Participants responded to the items on a 5-point Likert scale (1 = *Not at all confident*, 5 = *Very much confident*), with a higher scale score indicating higher trust in the government regarding its response to the COVID-19 pandemic.

Vaccination hesitancy, operationalized as vaccination delay, was calculated as the interval (in days) between actual vaccination dates and eligibility dates, accounting for demographics, health status, occupations, and infection history. Delay was treated as a continuous behavioral indicator of hesitancy, reflecting postponement of vaccination rather than outright refusal. Other details, including the data collection timeline, the timeline of COVID-19 vaccination policies in Hong Kong, and the graphical illustrations of the delay calculation, are shown in Figs. S3, S4, and S5, respectively.

### Analysis plan

We employed Cox proportional-hazard regression to examine the relationship between vaccine hesitancy factors and vaccination delay using the *coxph* function in the *survival* package in R. Covariates included trust, the 5C constructs, vaccination intention, and demographics. The actual vaccination hesitancy for the first and third doses, measured as delay, was modeled separately as outcomes in the two models. Although we hoped to obtain data from the participants closest to the time of vaccine rollouts, participants skipped rounds and rejoined at different times. Kaplan–Meier survival curves were used to visualize vaccination rates before and after vaccine mandates, with log-rank tests for significance.

We conducted SEM to examine the relationships between trust, 5C constructs, vaccination intention (from the earliest available data from the participants after the vaccine rollout), and actual vaccination hesitancy captured by delay (see Fig. S1), using the *lavaan* package in R^31^. Baseline demographics and the effect of vaccination mandate were controlled in the model. The SEM model examined is shown graphically in Fig. S1. We tested the measurement models and the structural model, and the goodness-of-fit was determined using the benchmark with root mean square error of approximation (RMSEA) and standardized root mean square residual (SRMR) below 0.08^32^ and fit indices, including the comparative fit index (CFI) and Tucker-Lewis Index (TLI) above 0.90^33^. While factor loadings and measurement model parameters were estimated, they are not presented in this manuscript, as the primary focus of the study was not on testing the psychometric properties of the scales but on modeling their influence on behavioral outcomes.

As we observed non-normal distributions in our delay outcomes (bimodal for the first dose, Poisson for the third dose, see Table 1), we performed the mixture SEM to capture the bimodality in first dose delay and sqrt-transformed the third dose delay to address positive skewness. The resulting latent class membership is represented in the SEM output as the “Cluster” variable. This does not represent a grouping variable in the conceptual model but rather denotes subpopulation classification generated by the mixture modeling process.

**Table 1:** Key demographics of the subjects

	First dose (*n* = 328)		Third dose (*n* = 477)	
	*n*	%	*n*	%
Age (years)	Mean = 35.50	SD = 11.63	Mean = 38.01	SD = 11.73
18–24	58	17.68%	45	9.43%
25–34	122	37.20%	175	36.69%
34–44	84	25.61%	132	27.67%
45–54	34	10.37%	76	15.93%
55–64	26	7.93%	36	7.55%
65+	4	1.22%	13	2.73%
Sex				
Male	117	35.67%	159	33.33%
Female	211	64.33%	318	66.67%
Days elapsed in taking the dose since the vaccine rollout	Mean = 184.50	SD = 130.01	Mean = 87.00	SD = 77.21
0–90 days	83	25.31%	322	67.51%
91–180 days	129	39.33%	105	22.01%
181–270 days	11	3.35%	32	6.71%
270–360 days	74	22.56%	12	2.52%
>360 days	31	9.45%	6	1.26%
	Mean	SD	Mean	SD
Age-specific duration of the first dose vaccination delay (days)				
18–24	136.78	(124.67)	93.62	(87.11)
25–34	187.77	(120.36)	93.25	(77.97)
34–44	180.79	(130.68)	75.94	(66.76)
45–54	205.88	(143.50)	83.57	(83.31)
55–64	240.27	(119.40)	94.11	(77.91)
65+	310.25	(216.40)	92.46	(91.95)
5C Constructs (1–7)				
Confidence	3.60	(1.47)	4.46	(1.33)
Complacency	3.26	(1.28)	3.54	(1.22)
Calculation	5.84	(0.95)	5.49	(1.02)
Constraint	2.99	(1.34)	3.17	(1.27)
Collective Responsibility	5.09	(1.14)	4.97	(1.11)
Institutional trust (5–25)	8.71	(4.16)	10.86	(4.74)

Since the difference in magnitude between the variables was large (three digits in the delay and a single digit in the predictors), we converted the unit of delay outcome from days to years by dividing the delay by 365 to avoid non-convergence in the SEM analysis. The same covariates were controlled in the SEM and Cox proportional-hazard regression models. While both models examined the same predictors and outcome, they served different analytic purposes: the Cox regression assessed direct associations with time-to-vaccination, whereas the SEM tested theory-driven mediation pathways linking institutional trust, psychological antecedents, and vaccination behavior. To control the effect of the differences in response timing, we included the round effect as a covariate in the Cox and SEM models. Experience of adverse effects in the first two doses was added as a covariate in the third dose model. This study was approved by the Survey Behavioral Research Ethics Committee of The Chinese University of Hong Kong (reference no. SBRE-21-0206) and performed in accordance with the Declaration of Helsinki.

## Results

### Demographic characteristics of the participants

Of 477 participants, their mean age was 38.01 (SD = 11.73, range = 21.00–69.50), and 66.67% were female (Table 1). For the first dose (*N* = 328, see Fig. S2), 28.35% received it within 100 days of eligibility, with a mean delay of 184.50 days (SD = 130.01), ranging from 1 to 590 days. Delay increased with age, from 136.78 days for ages 18–24 to 310.25 days for ages 65+. For the third dose (*N* = 475), 74.21% received it within 100 days of eligibility, with a mean delay of 87.00 days (SD = 77.21), ranging from 1 to 445 days. A U-shaped trend in delay with age was observed, from 93.62 days for ages 18–24, 75.94 days for ages 34–44, to 92.46 days for ages 65 + .

### Cox proportional-hazard regression model

**Fig. 1:**
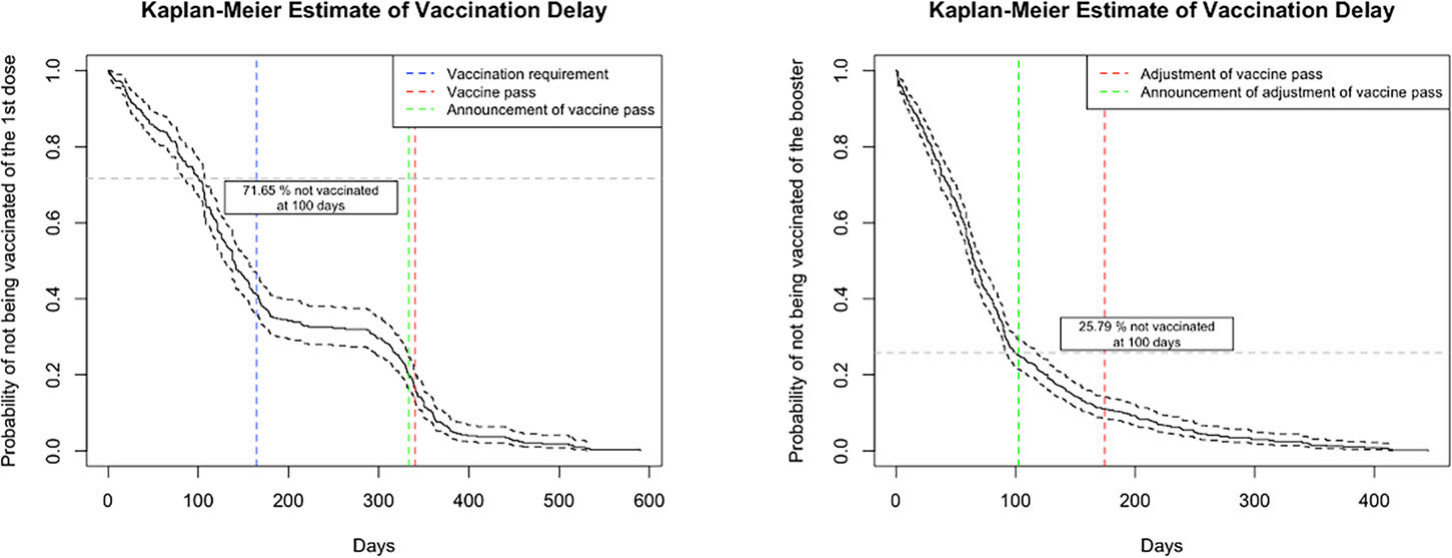
Kaplan–Meier survival curves of vaccination delay. Kaplan–Meier estimates of the probability of remaining unvaccinated over time (days since eligibility) are shown for **a** the first vaccine dose and **b** the third (booster) dose. The solid black line represents the Kaplan–Meier survival estimate, and the black dashed lines represent the 95% confidence intervals. Vertical reference lines indicate policy milestones: a blue dashed vertical line denotes implementation of the vaccination requirement; a red dashed vertical line denotes implementation of the vaccine pass; and a green dashed vertical line denotes announcement of the vaccine pass or announcement of its adjustment. The horizontal gray dashed line marks the estimated proportion remaining unvaccinated at 100 days, with the corresponding percentage displayed in the figure. The y-axis represents the probability of not being vaccinated, and the x-axis represents time in days. CD1 first vaccine dose, CD3 third (booster) vaccine dose.

**Fig. 2:**
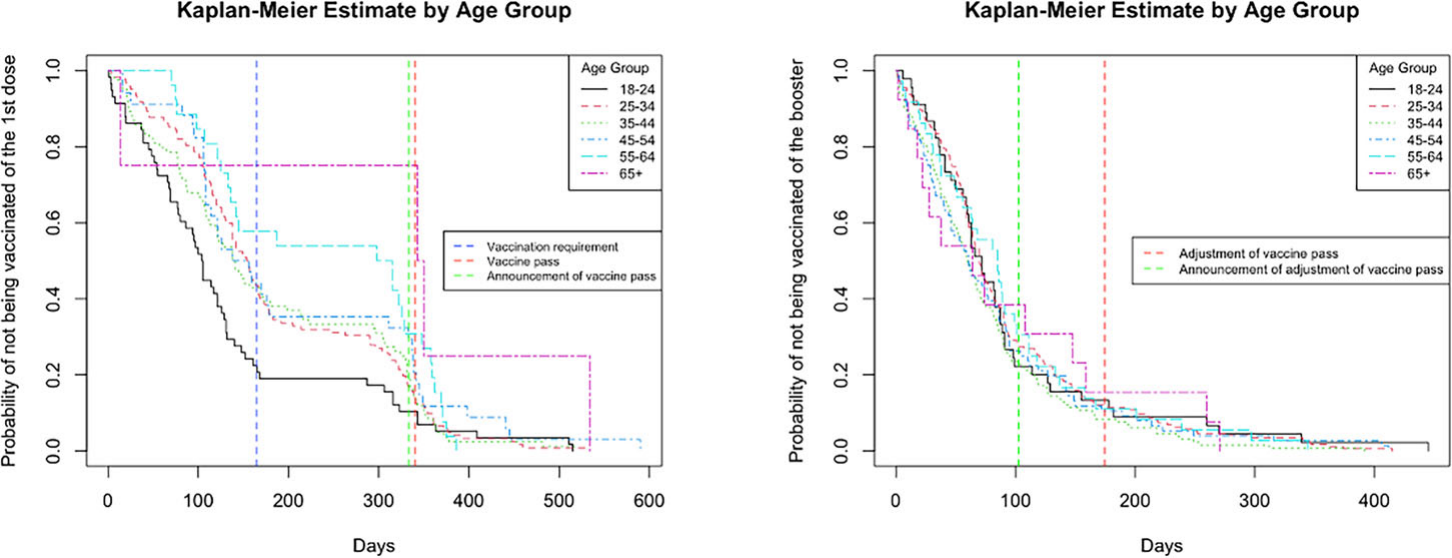
Kaplan–Meier survival curves of vaccination delay by age groups. Kaplan–Meier estimates of the probability of remaining unvaccinated over time (days since eligibility) stratified by age group are shown for **a** the first vaccine dose and **b** the third (booster) dose. The solid black line represents participants aged 18–24 years; the red dashed line represents participants aged 25–34 years; the green dotted line represents participants aged 35–44 years; the blue dot-dashed line represents participants aged 45–54 years; the cyan long-dashed line represents participants aged 55–64 years; and the magenta dashed line represents participants aged 65 years or older. Vertical reference lines indicate policy milestones: a blue dashed vertical line denotes implementation of the vaccination requirement; a red dashed vertical line denotes implementation of the vaccine pass; and a green dashed vertical line denotes announcement of the vaccine pass or announcement of its adjustment. The y-axis represents the probability of not being vaccinated, and the x-axis represents time in days. CD1 first vaccine dose, CD3 third (booster) vaccine dose.

Survival curves showed two turning points at the vaccination requirement and vaccine pass implementation (Fig. 1). Vaccination rates decreased significantly after the vaccination requirement (*p* < 0.001) and vaccine pass (*p* < 0.001). The slopes in the survival curve were steeper before the vaccination requirement and vaccine pass, reflecting participants rushing for vaccinations as the “deadlines” approached. As revealed by the age-specific survival curves (Fig. 2), older individuals were less responsive to the vaccination requirement but more responsive to the vaccine pass. In the model predicting the first dose delay (Table 2), intention predicted a 7.6% increase in vaccination rates (HR = 1.076, 95% CI: 1.029–1.125). Age groups 25+ were associated with 34.2–74.6% decreased rates of vaccination compared to ages 18–24 (although ages 35–44 were not significant). The responding round was associated with a decrease in the vaccination rate. Age-specific survival curves without adjustments for the eligibility date, accounting for demographics, health status, occupations, and infection history, are presented in Fig. S6.

**Table 2:** Cox proportional hazard regression model predicting vaccination delay

	First dose (*N* = 328)			Third dose (*N* = 477)		
Factors	HR	[95% CI]	*p*	HR	[95% CI]	*p*
Institutional trust	1.030	[0.997–1.064]	0.074	1.007	[0.982–1.031]	0.595
Confidence	1.036	[0.937–1.145]	0.495	1.044	[0.950–1.148]	0.366
Complacency	1.008	[0.883–1.149]	0.910	0.991	[0.906–1.084]	0.841
Collective responsibility	1.073	[0.923–1.247]	0.359	0.913	[0.813–1.026]	0.126
Calculation	1.024	[0.902–1.164]	0.710	1.125	[1.015–1.248]	0.025*
Constraint	1.017	[0.915–1.131]	0.758	0.951	[0.871–1.038]	0.260
Vaccination intention	1.076	[1.029–1.125]	0.001**	1.039	[1.001–1.078]	0.045*
Round	0.624	[0.561–0.695]	0.000***	0.941	[0.814–1.088]	0.414
Age: 18–24		Ref			Ref	
Age: 25–34	0.658	[0.474–0.914]	0.013*	1.007	[0.718–1.411]	0.968
Age: 35–44	0.758	[0.535–1.075]	0.120	1.209	[0.847–1.728]	0.296
Age: 45–54	0.440	[0.281–0.689]	0.000***	1.051	[0.717–1.542]	0.799
Age: 55–64	0.439	[0.271–0.711]	0.001**	0.947	[0.598–1.500]	0.817
Age: 65+	0.254	[0.086–0.749]	0.013*	0.936	[0.485–1.806]	0.843
Women	0.954	[0.749–1.215]	0.703	1.039	[0.853–1.265]	0.706
Experience of adverse effects				1.118	[0.890–1.405]	0.337

Results are based on two-tailed tests. Cox proportional hazard regressions were conducted using R.

***p < 0.001, **p < 0.01, *p < 0.05.

For the third dose, similar to the first dose, the slope of the survival curve flattened after the vaccine pass (*p* < 0.001 in the log-rank test). In the Cox model, calculation and vaccination intention were associated with 12.5% (HR = 1.125, 95% CI: 1.015–1.248) and 3.9% (HR = 1.039, 95% CI: 1.001–1.078) increased rates of vaccination, respectively.

### Structural equation modeling

Our SEM model of the first dose delay (Table 3; full table with estimates of covariates is shown in Table S2) achieved a good model fit (CFI = 0.959, TLI = 0.945, RMSEA = 0.047, SRMR = 0.049). Institutional trust predicted all 5 C constructs except complacency (confidence: estimate = 0.416, SE = 0.111, *p* < 0.001; complacency: estimate = 0.055, SE = 0.086, *p* = 0.358; collective responsibility: estimate = 0.168, SE = 0.077, *p* = 0.003; calculation: estimate = −0.186, SE = 0.066, *p* = 0.002; constraint: estimate = 0.227, SE = 0.094, *p* < 0.001). Women were associated with less confidence (estimate = −0.263, SE = 0.173, *p* < 0.001). Age group 25–34 was associated with more constraints (estimate = 0.155, SE = 0.210, *p* = 0.043), and 55–64 (estimate = −0.131, SE = 0.313, *p* = 0.041) and 65–74 with fewer constraints (estimate = −0.137, SE = 0.688, *p* = 0.017) than ages 18–24, respectively. Confidence positively (estimate = 0.518, SE = 0.150, *p* < 0.001) and complacency negatively (estimate = −0.264, SE = 0.204, *p* = 0.002) predicted intention, and intention, in turn, negatively predicted delay (estimate = −0.070, SE = 0.003, *p* = 0.012). The 25–34 (estimate = 0.061, SE = 0.023, *p* = 0.046) and 45–54 age groups (estimate = 0.074, SE = 0.031, *p* = 0.005) were associated with longer delay, with the 18–24 age group as the reference group. The responding round was associated with longer delay (estimate = 0.114, SE = 0.009, *p* < 0.001).

**Table 3:** Structural equation model predicting vaccination delay

	First dose (*N* = 328)^a^ CFI = 0.959, TLI = 0.945, RMSEA = 0.047, SRMR = 0.049				Third dose (*N* = 477)^b^ CFI = 0.943, TLI = 0.921, RMSEA = 0.050, SRMR = 0.056			
	Estimate^c^	SE	*z*	*p*	Estimate^c^	SE	*z*	*p*
Vaccination delay~								
Intention	−0.070	0.003	−2.510	0.012*	−0.123	0.004	−2.156	0.031*
Trust	−0.024	0.012	−0.885	0.376	−0.076	0.014	−1.326	0.185
Confidence	−0.014	0.009	−0.362	0.717	−0.056	0.012	−0.725	0.469
Complacency	−0.001	0.012	−0.015	0.988	0.001	0.014	0.014	0.988
Collective responsibility	−0.018	0.013	−0.439	0.661	0.175	0.018	1.897	0.058
Calculation	0.007	0.011	0.262	0.793	−0.149	0.014	−2.523	0.012*
Constraint	0.048	0.009	1.398	0.162	0.059	0.013	0.771	0.440
Intention~								
Confidence	0.518	0.150	6.427	0.000***	0.114	0.167	1.719	0.086
Complacency	−0.264	0.204	−3.112	0.002**	−0.030	0.186	−0.454	0.650
Collective responsibility	−0.114	0.227	−1.266	0.206	0.310	0.244	3.969	0.000***
Calculation	0.040	0.188	0.691	0.490	−0.082	0.189	−1.645	0.100
Constraint	0.048	0.163	0.635	0.525	−0.076	0.179	−1.178	0.239
Trust	0.065	0.210	1.079	0.280	0.236	0.191	4.917	0.000***
Confidence~								
Trust	0.416	0.111	6.995	0.000***	0.388	0.083	7.418	0.000***
Complacency~								
Trust	0.055	0.086	0.920	0.358	0.020	0.080	0.350	0.726
Collective responsibility~								
Trust	0.168	0.077	3.021	0.003**	0.211	0.071	3.792	0.000***
Calculation~								
Trust	−0.186	0.066	−3.066	0.002**	−0.138	0.056	−2.590	0.010*
Constraint~								
Trust	0.227	0.094	3.852	0.000***	−0.017	0.079	−0.306	0.759

Results are based on two-tailed tests. Covariates include being women, round, and experience of adverse effects (for the Third dose model only). The structural equation models were conducted using R.

“~” indicates a regression path with the left-hand variable as the outcome.

***p < 0.001, **p < 0.01, *p < 0.05.

^a^ Because of the bimodal nature of the outcome variable, mixture modeling was used.

^b^ Because of the Poisson nature of the outcome variable, the outcome variable was sqrt-transformed.

^c^ Standardized estimates with respect to the observed and latent variables.

An acceptable model fit was obtained for the third dose model (CFI = 0.943, TLI = 0.922, RMSEA = 0.050, SRMR = 0.056). Institutional trust positively predicted confidence (estimate = 0.388, SE = 0.083, *p* < 0.001) and collective responsibility (estimate = 0.211, SE = 0.071, *p* < 0.001) and negatively predicted calculation (estimate = −0.138, SE = 0.056, *p* = 0.010). Collective responsibility (estimate = 0.310, SE = 0.244, *p* < 0.001) and institutional trust (estimate = 0.236, SE = 0.191, *p* < 0.001) positively predicted intention. Intention (estimate = −0.123, SE = 0.004, *p* = 0.031) predicted shorter delay.

As sensitivity analyses, we conducted Cox and SEM analyses without data after the implementation of the vaccine pass (see Supplementary Materials, Tables S3 and S4). Results were similar whether or not the post-vaccine pass data were included.

## Discussion

Hong Kong was among the most successful regions in containing COVID-19 in the early phase of the pandemic, largely due to the population’s high adoption of non-pharmaceutical interventions such as social distancing, mask-wearing, and hand hygiene practices shaped by collective memories of the SARS outbreak^34^. However, this initial success may have reduced the perceived urgency of vaccination and contributed to vaccine hesitancy during the severe omicron wave in the later stage^35–37^. Comparing the vaccination coverage within 100 days after the launch of the vaccination program^38^ (see also the Supplementary Materials for details), Hong Kong had lower coverage (20.41%) than other developed regions like Israel (55.66%), the United Kingdom (38.90%), and the United States (29.04%). Despite having a more widespread outbreak, Israel maintained a lower mortality rate of COVID-19 than the United Kingdom and the United States^38^, the earliest batch of countries that launched vaccination programs, underscoring the importance of quick initial vaccination coverage amid a pandemic in suppressing the mortality rate and protecting vulnerable populations.

High vaccination coverage alone is insufficient without timely immunization to ensure effective protection against vaccine-preventable diseases^39^. Coverage reflects the eventual proportion vaccinated, whereas timeliness determines whether individuals are protected when disease risk is highest. Studies have shown that delayed vaccination can diminish herd protection and increase morbidity and mortality, even in populations that eventually achieve high coverage^40,41^. This was apparent in Hong Kong, where high third-dose coverage (89% as of 31 December 2022^38^) was accompanied by slow initial vaccination and a high mortality among older adults (aged 55+)^42^.

Vaccination hesitancy reflects societal responses to public health policies. Since July 2021, many countries have implemented policies to promote timely vaccination coverage^43^. Nine countries mandated vaccinations for the entire adult population, while 46 countries targeted specific groups. Regions like Hong Kong introduced “vaccine pass” policies, requiring vaccination certificates for entry into non-essential premises. Our data show a marked increase in vaccination rates before the vaccine pass, suggesting that its announcement and related public discussion encouraged more timely uptake. While such policies can effectively increase vaccination rates, their long-term success depends on maintaining public trust and avoiding perceptions of coercion^44^.

Delay should be considered alongside coverage to evaluate the success of vaccination programs. It directly measures delay in acceptance, a key but often-overlooked component of hesitancy^12^. Unlike self-reported attitudes or intentions, which are typically used in vaccine hesitancy literature^45^, delay captures real-world vaccination decisions, providing a clearer picture of hesitancy. Conceptually, delay reframes vaccine hesitancy as a dynamic process rather than a static attitude by incorporating a temporal dimension, thereby strengthening its relevance for behavioral public health research. Longer delay poses a greater risk for vulnerable populations^40,41,46^, serving as a critical warning indicator for public health policies. Such behavioral metrics allow more timely and effective policies during critical stages of a pandemic. Comparing populations with similar coverage but differing latencies helps policymakers pinpoint and address underlying barriers to vaccination.

Institutional trust significantly shapes vaccination intentions and behaviors^12,47^. Our study found that trust became increasingly important in predicting the vaccination delay as the campaign progressed, especially for the third dose. Early decisions were influenced by uncertainty over vaccine efficacy and side effects, whereas later uptake was driven more by prior experiences and contextual factors, underscoring the importance of trust in the later stages of a vaccination campaign^48,49^.

Our findings point to distinct intervention priorities across different phases of vaccine rollout, echoing our earlier work^5^. Strengthening confidence, a consistent predictor of intention, should remain central and can be supported through transparent messaging about vaccine safety, endorsements from trusted figures, and responsive communication. Addressing complacency is especially important early on through risk communication that highlights personal vulnerability. For booster uptake, promoting collective responsibility can foster social motivation, while simplifying vaccine information may reduce the effects of calculation, which can delay action. Although constraints did not directly predict outcomes, maintaining flexible access, such as walk-in hours or mobile clinics, remains important for timely uptake.

Transparency in communication is essential for maintaining public confidence in the vaccination program^50^. Providing scientifically credible and clear information about vaccine development and safety helps counter misinformation and builds trust^51,52^. Social media can also be used to create participatory dialogs^53^, while community engagement should be guided by culturally sensitive approaches^51,52^. Involving trusted community leaders, scientists, and researchers can further strengthen trust and promote timely vaccination^51,52^. Beyond simply delivering information, genuine community engagement that incorporates empathic listening and open, two-way dialog is crucial for understanding people’s concerns and fostering trust.

Despite the potential implications of this work, limitations should be noted. Reliance on self-report data raises concerns about response bias. Instances of negative vaccination delay, where participants reported vaccination dates earlier than eligibility, suggest recall bias in vaccination dates and/or demographics. Additionally, social desirability bias may have influenced vaccine-related responses, especially when being vaccine-hesitant could have been seen as unethical during the pandemic, leading to over-reporting positive attitudes and intentions. Another limitation involves inconsistent time gaps between the vaccination dates and the last data collection before vaccination across participants. Some participants missed rounds, and the intervals between rounds were inconsistent (see Table S1 and Figs. S2, S3). Although we controlled for the round effect in our analyses, we could not fully capture the impact of all significant events in the study period. Future research could benefit from supplementing self-reported data with more objective evidence, such as implicit attitude measures^5^ and medical records, to reduce social desirability bias in vaccine-related items and eliminate recall bias of vaccination dates.

After the 2003 SARS epidemic, Hong Kong demonstrated remarkable resilience by swiftly adopting non-pharmaceutical measures during the COVID-19 pandemic^29^. However, building trust in COVID-19 vaccines proved challenging, leading to a slow initial uptake. For future epidemics or pandemics, healthcare authorities should adopt flexible, empathetic strategies that are culturally attuned and built on genuine community engagement to encourage timely vaccination.

In summary, this study underscores two public health implications for addressing vaccination hesitancy and, thus, improving future pandemic preparedness: (1) the importance of considering vaccination delay as a metric for vaccine hesitancy to understand the complex interplay between evolving policies, pandemic progression, and vaccination decision-making and (2) the role of institutional trust in shaping attitudes toward vaccines and the extent of hesitancy, alongside the impact of public health interventions. SEM models reveal the complex interplays among institutional trust, 5 C constructs, and vaccination hesitancy within the theory of planned behavior. Higher trust consistently predicted confidence, collective responsibility, and lower calculation, resulting in higher intentions and shorter delays. Interestingly, in the third dose model, individuals vaccinated later showed higher intentions but lower confidence and collective responsibility, along with higher complacency and constraints, suggesting other factors, like vaccination policies, influenced decisions.

## Supplementary information

Supplementary Materials

## Data Availability

The datasets used or analyzed during the current study, including the source data for Figs. 1–2 and Tables 1–3, are available from the corresponding authors via GitHub (https://github.com/kkokwok/com_med/tree/main/26_vaccination_delay) and are openly accessible to the public.

## Code availability

Data manipulations and other analyses were conducted using R version 4.23 (https://www.r-project.org). The underlying statistical code for the current study is available from the corresponding authors via GitHub (https://github.com/kkokwok/com_med/tree/main/26_vaccination_delay) and is openly accessible to the public.
