# Supplementary material for: Institutional trust and vaccination delay as key metrics for vaccination rollout success
Source: Commun Med (Lond). 2026 Apr 29;6:376. doi: 10.1038/s43856-026-01597-4 (PMC13333957; doi:10.1038/s43856-026-01597-4)
Supplement: Supplementary file 1 — Supplementary Materials [file 43856_2026_1597_MOESM1_ESM.pdf]

SUPPLEMENTARY MATERIALS

5C AND THE OTHER FACTORS PREDICTING VACCINE HESITANCY

The 5C psychological antecedents of vaccination, Confidence, Complacency, Constraints, Calculation, and Collective Responsibility, offer a structured framework to account for a wide range of individual, social, and contextual determinants that have been shown to influence vaccine uptake [1]. Rather than modeling each potential predictor separately, we include the 5C constructs to parsimoniously capture the psychological pathways through which diverse factors, such as prior infection experience, caregiving burden, occupational status, educational background, and perceived social duty, shape vaccination behavior. In doing so, the 5C framework serves as a proxy for many external influences highlighted in the literature, while allowing us to model their psychological relevance for decision-making regarding vaccine timing. Below, we elaborate on how each construct reflects specific influences relevant to COVID-19 vaccine delay, with supporting references.

Confidence reflects individuals' beliefs about vaccine safety, effectiveness, and the underlying scientific rationale. Hesitancy may arise from personal doubts about how well vaccines protect against infection, how long the protection lasts, or whether side effects are accurately reported [2]. These doubts may be shaped by past vaccination experiences, peer accounts of adverse reactions, or general skepticism toward medical interventions. Confidence is also influenced by trust in the scientific process, including how vaccines are tested, approved, and monitored, especially when new technologies, such as mRNA-based vaccines, are involved [3].

Complacency captures the extent to which individuals perceive the disease to be a serious threat. People who have been infected by COVID-19 but had only mild or asymptomatic infections often downplay the risks of reinfection or assume they have sufficient natural immunity, thus deprioritizing vaccination [4]. This mindset is particularly relevant in the context of booster uptake, where prior experience heavily informs perceived need. Even among those who have not been infected, perceptions shaped by misinformation, peer narratives, or broader community sentiment about COVID-19 severity can result in lowered urgency to vaccinate. Rather than opposing vaccination, these individuals may simply not see it as immediately necessary.

Constraints refer to the structural and situational barriers that hinder an individual's ability to get vaccinated despite their willingness. These include factors such as long working hours, inflexible shift-based jobs (e.g., service workers, delivery personnel), or precarious employment that discourages taking time off [5]. Caregiving responsibilities, such as taking care of young children or elderly family members, also act as major constraints when vaccination sites or hours are not accessible [6]. In densely populated or underserved areas, distance to vaccination centers and public transport availability further compound these constraints.

Calculation reflects the extent to which individuals actively deliberate and seek information before making vaccination decisions. Those with higher levels of education or health literacy tend to evaluate the pros and cons of different vaccine options, often comparing efficacy rates, side effects, clinical trial data, and country-of-origin considerations. While this deliberative process may reflect engagement and autonomy, it often leads to vaccination delay as individuals wait for more data or consensus before making a decision. Importantly, this group is not vaccine-resistant but rather cautious, sometimes excessively so [7]. These behaviors are not fully explained by demographic profiles but are embodied in the calculation domain.

Collective responsibility represents the motivation to vaccinate not solely for personal benefit, but to protect others. Individuals living with high-risk family members, such as the immunocompromised, the elderly, or young children, often perceive vaccination as a social duty [8]. Similarly, those working in healthcare or high-contact settings may feel a professional or ethical obligation to be vaccinated early to minimize risk to others [9]. This sense of obligation can override personal hesitation or risk aversion, driving earlier uptake even among those with concerns. In contrast, individuals who do not feel socially accountable, or whose networks do not reinforce these values, may delay without perceiving ethical implications. This dimension helps capture social contextual drivers that are not reducible to personal intention alone.

METHODOLOGY

Study Timeline

We recruited and distributed the online survey link of the platform *SurveyMonkey* to the cohort participants with the help of the district councilors through their usual communication routes to the residents in their service districts. The cohort included adults aged 18+ residing in Hong Kong for at least five days a week on average in the last month. Participants were compensated with dining or shopping vouchers as a token of appreciation upon completing the online survey in each round.

The thirteen rounds of the cohort study covered the different stages of the COVID-19 pandemic in Hong Kong (details shown in Appendices Table S1 and Figure S3), including the first wave (R1), the second wave (R2 and R3), the third wave (R4 and R5), the fourth wave (R6 and R7), a dormant period after the fourth wave and launching period of the vaccination program in Hong Kong (R8 and R9), the initial implementation stage of vaccination requirement in the government and global corporations (R9), the fifth wave of the pandemic (R10 to R13), and the enforcement of the vaccine pass (R11 to R13).

Cases from individuals who claimed an earlier-than-available date of vaccination based on their reported demographics and infection history, which reflected mistaken reporting of vaccination dates, demographics, and/or infection history in the survey, would be discarded.

Further Information about the Calculation of Vaccination Delay

Vaccine Rollout

The vaccine rollout date in Hong Kong was 26 February 2021[10], while the booster was available on 11 November 2021 [11]. Given the limited availability of the vaccines at the early stage of the vaccine rollout, high-risk and vulnerable populations were prioritized, and the vaccines were later made available to the general public. Throughout the study period, participants retained considerable autonomy over the timing of their vaccination. From the launch of the vaccination campaign in February 2021 until the announcement of the first mandates in August 2021, uptake was entirely voluntary. Even after the government introduced mandatory vaccination policies, such as requirements for civil servants or the implementation of the vaccine pass, these policies were rolled out in phases and included grace periods, during which individuals could still choose when to comply. This allowed for behavioral variation in vaccine timing, even under regulatory incentives. Our measurement of vaccination delay thus captures not only voluntary early uptake but also individual responsiveness within policy-constrained

settings. Also, recovered individuals were recommended to take the vaccines after a buffer period. The rollout expansion timeline is shown in Supplementary Materials Figure S4.

During the COVID-19 vaccination campaign in Hong Kong, residents were given a choice between two main vaccine types: the BioNTech mRNA vaccine (Comirnaty) and the inactivated Sinovac vaccine (CoronaVac). The government also designated multiple community vaccination centers and private clinics as providers, allowing individuals to choose both vaccine type and location. While these contextual elements may influence individual preferences and decisions, our model focuses on psychological determinants. Constructs such as confidence and institutional trust can proxy or reflect individual perceptions of vaccine safety, efficacy, and system-level credibility associated with different options. It is also important to note that health authorities recommend individuals maintain the same vaccine type for their initial two-dose series. As our previous study [12] indicates, only 5.2% of individuals switched to a different vaccine type for their third (booster) dose, suggesting relatively limited behavioral variability in vaccine-type switching during the booster rollout.

Vaccine Mandates

The HKSAR Government announced the vaccination requirement for all civil servants on 2 August 2021, and in effect on 1 September 2021 [13]. Major corporations in Hong Kong have followed and implemented the same requirement for their employees on the same date. The implementation of the vaccine pass (for the first two doses of the COVID-19 vaccines) was announced on 17 February 2022 and in effect on 24 February 2022 [14]. The adjustment of the vaccine pass that included the third dose in the mandate was announced on 20 March 2022 and in effect on 31 May 2022 [15].

Comparison of Vaccination

Vaccination programs were launched on 26 February 2021 in Hong Kong [16]; on 8 December 2020 in the United Kingdom [17]; on 14 December 2020 in the United States [18]; on 20 December 2020 in Israel [19]; on 30 December 2020 in Singapore [20]; and on 17 February 2021 in Japan [21].

Making reference to the Our World in Data dataset [22], only 20.41% of the Hong Kong population was vaccinated in the first 100 days since the launch of the vaccination program (i.e., as of 6 June 2021, similar to our findings, 28.35%), a rate lower than that observed in Israel (55.66% as of 30 March 2021), the United Kingdom (38.90% as of 18 March 2021) and the United States (29.04% as of March 24, 2021). Compared to other high-income Asian countries, Hong Kong's rate was similar to Singapore's (20.85% as of 9 April 2021) and higher than Japan's (8.99% as of 28 May 2021). Comparing the earliest countries that launched their vaccination programs, Israel managed to keep a lower mortality rate of COVID-19 than the United States and the United Kingdom (7-day rolling average of daily confirmed death as of 28 March 2021: Israel = 15.95, United States = 33.37, United Kingdom = 34.15) [22], despite having a more widespread outbreak (7-day rolling average of daily confirmed cases as of 28 March 2021: Israel = 2200.70, United States = 1356.37, United Kingdom = 832.99), highlighted the importance of quick initial vaccination coverage amid a pandemic in suppressing the mortality rate and protecting the vulnerable populations.

Specification of the SEM Models

The structural equation model comprised a measurement component and a structural component. The measurement component defined latent variables for institutional trust and each of the five psychological antecedents of vaccination (confidence, complacency, constraints, calculation, and collective responsibility). Each construct was measured using three observed items from the validated 5C scale.

The structural component followed a mediation pathway in which institutional trust predicted each of the five psychological antecedents, which in turn predicted vaccination intention, and ultimately vaccination delay. This structure was grounded in the Theory of Planned Behavior and aimed to capture both direct and indirect pathways linking institutional-level attitudes with vaccine uptake behavior.

To reflect the conceptual interrelatedness of the five psychological antecedents—each representing a facet of vaccine hesitancy—we included residual covariances between their latent variables. These covariances accounted for shared variance not explained by the structural pathways, consistent with theoretical expectations and previous applications of the 5C model. No residual covariances were estimated between item-level errors.

The model included institutional trust as the primary exogenous variable. Age and gender were added as covariates. While other sociodemographic and contextual factors were not explicitly modeled, practical barriers such as work schedule or access are conceptually embedded within the "constraints" construct of the 5C framework."

RESULTS

Sensitivity Analysis

Cox Proportional-Hazard Regression Model (Excluding post-mandate data)

The results of the Cox proportional-hazards regression models, excluding data reported after the implementation of the vaccine pass, are presented in Table S2. There were 286 cases retained in the Cox proportional-hazards regression model predicting vaccination delay of the first dose, as in the main analysis. Results in the sensitivity analysis were similar to those in the main analysis. In the model predicting the delay administering the first dose, vaccination intention was associated with 8.1% increased rate of vaccination (HR = 1.081, 95% CI: 1.029-1.135), age groups of 25-34, 35-44, 45-54, 55-64 and 65+ were associated with 54.9% (HR = 0.451, 95% CI: 0.319-0.638), 49.6% (HR = 0.504, 95% CI: 0.348-0.731), 55.4% (HR = 0.446, 95% CI: 0.277-0.719), 81.9% (HR = 0.181, 95% CI: 0.102-0.323), and 90.5% (HR = 0.027, 95% CI: 0.027-0.340) decreased rate of vaccination, respectively, compared to the age group of 18-24.

We had 341 cases in the Cox proportional-hazard regression model predicting the vaccination delay of the booster without post-mandate data. Calculation and vaccination intention were associated with 13.9% (HR = 1.139, 95% CI: 1.004-1.292) and 4.8% (HR = 1.048, 95% CI: 1.005-1.094) increased rates of vaccination, respectively, while collective responsibility was associated with 18.5% (HR = 0.815, 95% CI: 0.707-0.940) decreased rate of vaccination.

Structural Equation Modeling (Excluding data reported after the implementation of the vaccine pass)

The results of the structural equation models (SEM) without post-mandate data are shown in Table S3. We achieved a good model fit for the SEM model predicting vaccination delay of the first dose (CFI = 0.964, TLI = 0.951, RMSEA = 0.044, SRMR = 0.050). In the model predicting the delay administering the first dose, institutional trust predicted all of the 5C constructs except for complacency. Being women was associated with less confidence (estimate = -0.268, SE = 0.187, $p < 0.001$). The age group of 65-74 was associated with fewer constraints than the age group of 18-24. Confidence positively (estimate = 0.532, SE = 0.147, $p < 0.001$) and complacency negatively (estimate = -0.293, SE = 0.206, $p = 0.001$) predicted vaccination intention, and vaccination intention, in turn, negatively predicted vaccination delay (estimate = -0.078, SE = 0.003, $p = 0.024$). The age groups 25-34, 45-54, and 55-64 were associated with longer delay than the 18-24 age group.

An acceptable model fit was obtained for the SEM model predicting vaccination delay of the third dose (CFI = 0.935, TLI = 0.911, RMSEA = 0.053, SRMR = 0.061). Institutional trust positively predicted confidence (estimate = 0.331, SE = 0.091, $p < 0.001$) and collective responsibility (estimate = 0.192, SE = 0.079, $p = 0.004$). Being women was associated with less confidence (estimate = -0.141, SE = 0.139, $p = 0.010$). Confidence (estimate = 0.179, SE = 0.195, $p = 0.015$), collective responsibility (estimate = 0.269, SE = 0.268, $p = 0.001$), and institutional trust (estimate = 0.267, SE = 0.209, $p < 0.001$) positively predicted vaccination intention. Institutional trust alone predicted shorter vaccination delay (estimate = -0.173, SE = 0.003, $p = 0.012$).

REFERENCES

1. Betsch, C. *et al.* Beyond confidence: Development of a measure assessing the 5C psychological antecedents of vaccination. *PLOS ONE* **13**, e0208601 (2018).
2. Larson, H. J., Cooper, L. Z., Eskola, J., Katz, S. L. & Ratzan, S. Addressing the vaccine confidence gap. *The Lancet* **378**, 526–535 (2011).
3. Arsenault, C. *et al.* The role of health systems in shaping vaccine decisions: Insights from Italy, Mexico, the United Kingdom, and the United States. *Vaccine* **54**, 127134 (2025).
4. Huang, Y. *et al.* COVID-19 Vaccine Hesitancy Among Patients Recovered From COVID-19 Infection in Wuhan, China: Cross-Sectional Questionnaire Study. *JMIR Public Health and Surveillance* **9**, e42958 (2023).
5. Cascini, F. *et al.* Social media and attitudes towards a COVID-19 vaccination: A systematic review of the literature. *eClinicalMedicine* **48**, 101454 (2022).
6. Reverte, V. *et al.* Does a correlation exist between delayed vaccination and a decreased vaccine confidence? *Hum Vaccin Immunother* **20**, 2419750.
7. Espejo, B. & Checa, I. Do gender and educational level predict vaccination? The mediating role of attitudes towards vaccines and fear of COVID-19. https://doi.org/10.1111/spc3.12879 (2023) doi:10.1111/spc3.12879.
8. Rozek, L. *et al.* Understanding Vaccine Hesitancy in the Context of COVID-19: The Role of Trust and Confidence in a Seventeen-Country Survey. *Int J Public Health* **66**, 636255 (2021).
9. Wu, J., Chen, C. H., Wang, H. & Zhang, J. Higher Collective Responsibility, Higher COVID-19 Vaccine Uptake, and Interaction with Vaccine Attitude: Results from Propensity Score Matching. *Vaccines (Basel)* **10**, 1295 (2022).
10. Centre for Health Protection. Statistics on Government COVID-19 Vaccination Programme. https://www.chp.gov.hk/en/features/106989.html (2024).
11. HKSAR Government. Third dose COVID-19 vaccination arrangements for persons under certain groups. https://www.info.gov.hk/gia/general/202111/03/P2021110300536.htm (2021).

12. Kwok, K. O. *et al.* The way forward to achieve high COVID-19 vaccination and revaccination coverage in a city amid a period of tranquility. *Frontiers in Public Health* (2022).
13. HKSAR Government. Vaccination of Government and Key Public Service Sector Employees. (2021).
14. HKSAR Government. Public urged to get ready for 'vaccine pass' with latest version of 'LeaveHomeSafe' app. https://www.info.gov.hk/gia/general/202202/17/P2022021700286.htm (2022).
15. HKSAR Government. Government adjusts vaccination requirements of Vaccine Pass. https://www.info.gov.hk/gia/general/202203/20/P2022032000438.htm (2022).
16. HKSAR Government. Government announces 2019 COVID-19 Vaccination Programme (with video). https://www.info.gov.hk/gia/general/202102/18/P2021021800767.htm?fontSize=1 (2021).
17. Baraniuk, C. Covid-19: How the UK vaccine rollout delivered success, so far. *BMJ* **372**, n421 (2021).
18. U.S. Department of Health and Human Services. COVID-19 Vaccines. https://www.hhs.gov/coronavirus/covid-19-vaccines/index.html (2023).
19. Israel Ministry of Health. 280,000 Vaccinated in the First Week of the 'Give a Shoulder' Campaign. *GOV.IL* https://www.gov.il/en/departments/news/27122020-01 (2020).
20. Government of Singapore. What you should know about the COVID-19 vaccine. *A Singapore Government Agency Website* https://www.gov.sg/article/what-you-should-know-about-the-covid-19-vaccine (2021).
21. Kayano, T. *et al.* Evaluating the COVID-19 vaccination program in Japan, 2021 using the counterfactual reproduction number. *Sci Rep* **13**, 17762 (2023).
22. Roser, M., Ritchie, H., Ortiz-Ospina, E. & Hasell, J. Coronavirus Pandemic (COVID-19). *OurWorldInData.org* https://ourworldindata.org/coronavirus (2020).
23. Lazarus, J. V. *et al.* Revisiting COVID-19 vaccine hesitancy around the world using data from 23 countries in 2021. *Nat Commun* **13**, 3801 (2022).

24. HKSAR Government. Government expands scope of priority groups and opens more CVCs (with photos/video). https://www.info.gov.hk/gia/general/202103/08/P2021030800738.htm?fontSize=1 (2021).

25. HKSAR Government. Vaccination priority groups to be expanded to cover people aged 30 or above. https://www.info.gov.hk/gia/general/202103/15/P2021031500626.htm?fontSize=1 (2021).

26. HKSAR Government. COVID-19 Vaccination Programme opens to persons aged 16 or above. https://www.info.gov.hk/gia/general/202104/15/P2021041500565.htm?fontSize=1 (2021).

27. HKSAR Government. Government extends third dose COVID-19 vaccination arrangements. https://www.info.gov.hk/gia/general/202111/18/P2021111800310.htm?fontSize=1 (2021).

28. HKSAR Government. Further expansion of COVID-19 vaccination arrangements from January 1. https://www.info.gov.hk/gia/general/202112/24/P2021122400509.htm?fontSize=1 (2021).

29. HKSAR Government. Government announces arrangement on COVID-19 vaccinations for persons with previous COVID-19 infection. https://www.info.gov.hk/gia/general/202105/21/P2021052100730.htm?fontSize=1 (2021).

30. HKSAR Government. Persons aged 18 to 59 may choose to receive fourth dose of COVID-19 vaccine. https://www.info.gov.hk/gia/general/202205/21/P2022052000831.htm?fontSize=1 (2022).

31. HKSAR Government. Announcement of appointment arrangements for shortened intervals between COVID-19 vaccine doses. https://www.info.gov.hk/gia/general/202203/04/P2022030400691.htm?fontSize=1 (2022).

32. HKSAR Government. Announcement of latest arrangements on COVID-19 vaccination. https://www.info.gov.hk/gia/general/202203/18/P2022031800485.htm?fontSize=1 (2022).

Table S1. The period covered, sample sizes of each data collection rounds, and demographics

Round	Period	# of days	N	Events	Mean daily new cases in Hong Kong (SD) *	Mean age (SD)	% of female
1	2020-01-23 – 2020-02-15	24	1712	1st wave	23.00 (17.90)	34.37 (11.96)	68.58%
2	2020-03-06 – 2020-04-14	40	620	2nd wave	22.68 (17.97)	35.41 (11.72)	69.52%
3	2020-05-08 – 2020-06-16	40	516	2nd wave	1.70 (2.66)	35.33 (11.72)	69.19%
4	2020-07-15 – 2020-08-07	24	455	3rd wave	98.71 (31.79)	34.77 (11.36)	70.11%
5	2020-08-08 – 2020-09-15	39	420	3rd wave	26.59 (21.38)	35.09 (11.69)	68.57%
6	2020-10-19 – 2020-12-01	44	410	4th wave	26.25 (32.78)	35.96 (11.81)	66.83%
7	2020-12-12 – 2021-01-17	37	363	4th wave	58.92 (21.18)	35.33 (11.70)	66.12%
8	2021-04-07 – 2021-05-23	47	308	Dormant period Vaccination program launched	6.40 (6.01)	34.79 (11.92)	67.21%
9	2021-09-20 – 2021-11-02	44	237	Dormant period Vaccination program launched	4.93 (4.87)	34.99 (11.72)	62.87%
10	2021-12-06 – 2022-01-17	43	843	5th wave Booster requirement	13.65 (11.47)	38.96 (12.28)	62.63%
11	2022-02-01 – 2022-02-22	22	982	5th wave Vaccine passport	2380.77 (2481.33)	37.24 (12.02)	68.84%
12	2022-04-14 – 2022-05-17	34	799	5th wave Vaccine passport	434.50 (209.17)	38.42 (11.87)	66.46%
13	2022-08-29 - 2022-09-17	20	545	5th wave Vaccine passport	9310.05 (1101.75)	38.39 (11.36)	66.42%

* Data from The World in Data [23]

Table S2. Structural equation model predicting vaccination delay

	First dose (N = 328)[a] CFI = .959, TLI = .945, RMSEA = .047, SRMR = .049				Third dose (N = 477)[b] CFI = .943, TLI = .921, RMSEA = .050, SRMR = .056			
	Estimate†	SE	z	p	Estimate†	SE	z	p
Vaccination delay ~								
Intention	-0.070	0.003	-2.510	0.012*	-0.123	0.004	-2.156	0.031*
Trust	-0.024	0.012	-0.885	0.376	-0.076	0.014	-1.326	0.185
Confidence	-0.014	0.009	-0.362	0.717	-0.056	0.012	-0.725	0.469
Complacency	-0.001	0.012	-0.015	0.988	0.001	0.014	0.014	0.988
Collective responsibility	-0.018	0.013	-0.439	0.661	0.175	0.018	1.897	0.058
Calculation	0.007	0.011	0.262	0.793	-0.149	0.014	-2.523	0.012*
Constraint	0.048	0.009	1.398	0.162	0.059	0.013	0.771	0.440
Cluster	0.801	0.023	26.730	0.000***				
Age (25-34)	0.061	0.023	1.991	0.046*	-0.001	0.033	-0.014	0.989
Age (35-44)	0.021	0.024	0.703	0.482	-0.089	0.035	-1.149	0.251
Age (45-54)	0.074	0.031	2.792	0.005**	-0.038	0.039	-0.543	0.587
Age (55-64)	0.049	0.035	1.868	0.062	0.010	0.046	0.175	0.861
Age (65-74)	0.043	0.074	1.885	0.059	-0.004	0.065	-0.077	0.939
Women	-0.002	0.018	-0.083	0.934	-0.015	0.020	-0.325	0.746
Round	0.114	0.009	3.512	0.000***	0.068	0.015	1.373	0.170
Experience of adverse effects					-0.034	0.022	-0.716	0.474
Intention ~								
Confidence	0.518	0.150	6.427	0.000***	0.114	0.167	1.719	0.086
Complacency	-0.264	0.204	-3.112	0.002**	-0.030	0.186	-0.454	0.650
Collective responsibility	-0.114	0.227	-1.266	0.206	0.310	0.244	3.969	0.000***
Calculation	0.040	0.188	0.691	0.490	-0.082	0.189	-1.645	0.100
Constraint	0.048	0.163	0.635	0.525	-0.076	0.179	-1.178	0.239
Trust	0.065	0.210	1.079	0.280	0.236	0.191	4.917	0.000***
Age (25-34)	-0.072	0.400	-1.063	0.288	0.081	0.441	1.217	0.223
Age (35-44)	-0.073	0.428	-1.120	0.263	0.188	0.456	2.925	0.003**
Age (45-54)	-0.008	0.547	-0.141	0.888	0.219	0.510	3.737	0.000***
Age (55-64)	0.040	0.610	0.693	0.488	0.077	0.603	1.529	0.126
Age (65-74)	-0.006	1.320	-0.121	0.904	0.080	0.856	1.812	0.070
Women	0.036	0.315	0.680	0.497	-0.035	0.263	-0.891	0.373
Round	0.013	0.128	0.235	0.814	0.166	0.197	4.118	0.000***
Experience of adverse effects					-0.106	0.286	-2.684	0.007**
Confidence ~								
Trust	0.416	0.111	6.995	0.000***	0.388	0.083	7.418	0.000***
Age (25-34)	-0.011	0.234	-0.145	0.885	-0.049	0.210	-0.609	0.543

Table S2. Structural equation model predicting vaccination delay

Age (35-44)	-0.010	0.253	-0.140	0.889	-0.008	0.218	-0.098	0.922
Age (45-54)	0.079	0.318	1.251	0.211	-0.116	0.239	-1.679	0.093
Age (55-64)	-0.028	0.349	-0.460	0.646	-0.046	0.284	-0.770	0.441
Age (65-74)	0.081	0.769	1.473	0.141	0.049	0.408	0.936	0.349
Women	-0.263	0.173	-4.902	0.000***	-0.088	0.123	-1.925	0.054
Round	-0.141	0.064	-2.650	0.008**	-0.112	0.089	-2.432	0.015*
Experience of adverse effects					0.009	0.136	0.203	0.839
Complacency ~								
Trust	0.055	0.086	0.920	0.358	0.020	0.080	0.350	0.726
Age (25-34)	0.058	0.198	0.726	0.468	0.087	0.217	0.949	0.342
Age (35-44)	0.080	0.214	1.022	0.307	0.045	0.224	0.514	0.607
Age (45-54)	0.121	0.269	1.758	0.079	0.052	0.247	0.661	0.509
Age (55-64)	0.104	0.297	1.555	0.120	-0.036	0.292	-0.534	0.594
Age (65-74)	-0.073	0.652	-1.221	0.222	-0.070	0.419	-1.162	0.245
Women	0.089	0.144	1.539	0.124	0.063	0.126	1.203	0.229
Round	0.322	0.056	5.340	0.000**	0.187	0.094	3.439	0.001**
Experience of adverse effects					-0.003	0.139	-0.055	0.956
Collective responsibility ~								
Trust	0.168	0.077	3.021	0.003**	0.211	0.071	3.792	0.000***
Age (25-34)	-0.053	0.173	-0.721	0.471	0.039	0.186	0.437	0.662
Age (35-44)	-0.073	0.187	-1.013	0.311	0.042	0.193	0.492	0.623
Age (45-54)	-0.054	0.236	-0.857	0.391	0.042	0.212	0.544	0.586
Age (55-64)	-0.035	0.259	-0.574	0.566	0.036	0.254	0.543	0.587
Age (65-74)	0.084	0.576	1.519	0.129	0.076	0.363	1.308	0.191
Women	-0.006	0.127	-0.107	0.915	0.027	0.109	0.525	0.600
Round	-0.454	0.056	-7.207	0.000***	-0.192	0.081	-3.687	0.000***
Experience of adverse effects					-0.009	0.121	-0.173	0.863
Calculation ~								
Trust	-0.186	0.066	-3.066	0.002**	-0.138	0.056	-2.590	0.010*
Age (25-34)	0.007	0.148	0.092	0.927	0.081	0.149	0.947	0.343
Age (35-44)	0.042	0.159	0.535	0.593	-0.002	0.154	-0.023	0.982
Age (45-54)	0.031	0.201	0.444	0.657	0.031	0.169	0.422	0.673
Age (55-64)	0.053	0.220	0.799	0.424	0.111	0.202	1.744	0.081
Age (65-74)	0.086	0.486	1.441	0.150	0.050	0.289	0.895	0.371
Women	0.101	0.108	1.743	0.081	-0.011	0.087	-0.223	0.824
Round	-0.094	0.040	-1.617	0.106	0.023	0.063	0.468	0.640
Experience of adverse effects					0.078	0.096	1.557	0.119
Constraint ~								
Trust	0.227	0.094	3.852	0.000***	-0.017	0.079	-0.306	0.759

Table S2. Structural equation model predicting vaccination delay

Age (25-34)	0.155	0.210	2.019	0.043*	0.036	0.210	0.410	0.682
Age (35-44)	0.073	0.226	0.971	0.331	0.013	0.218	0.153	0.879
Age (45-54)	-0.006	0.284	-0.092	0.927	-0.045	0.239	-0.592	0.554
Age (55-64)	-0.131	0.313	-2.044	0.041*	-0.127	0.286	-1.925	0.054
Age (65-74)	-0.137	0.688	-2.387	0.017*	-0.096	0.409	-1.652	0.098
Women	0.021	0.152	0.385	0.700	0.032	0.123	0.638	0.523
Round	0.261	0.057	4.663	0.000***	0.068	0.089	1.328	0.184
Experience of adverse effects					0.036	0.136	0.698	0.485

***p < 0.001, **p < 0.01, *p < 0.05. [†]Standardized estimates with respect to the observed and latent variables

[a] Because of the bimodal nature of the outcome variable, mixture modelling was used.

[b] Because of the Poisson nature of the outcome variable, the outcome variable was sqrt-transformed.

Results are based on two-tailed tests.

Table S3. Cox proportional hazard regression model predicting vaccination delay (without post-mandate data)

	First dose (N = 286)			Third dose (N = 341)		
Factors	HR	[95% CI]	p	HR	[95% CI]	p
Institutional trust	1.034	[0.995-1.074]	0.084	1.015	[0.988-1.043]	0.279
Confidence	0.967	[0.869-1.077]	0.546	1.052	[0.941-1.177]	0.371
Complacency	0.980	[0.853-1.125]	0.771	0.922	[0.831-1.024]	0.129
Collective responsibility	0.969	[0.828-1.133]	0.693	0.815	[0.707-0.940]	0.005**
Calculation	0.998	[0.864-1.153]	0.981	1.139	[1.004-1.292]	0.044*
Constraint	0.976	[0.875-1.090]	0.670	1.015	[0.915-1.127]	0.775
Vaccination intention	1.081	[1.029-1.135]	0.002**	1.048	[1.005-1.094]	0.030*
Round	0.611	[0.530-0.703]	0.000***	1.052	[0.812-1.363]	0.703
Age: 18-24		Ref			Ref	
Age: 25-34	0.451	[0.319-0.638]	0.000***	1.072	[0.729-1.578]	0.724
Age: 35-44	0.504	[0.348-0.731]	0.000***	1.363	[0.911-2.039]	0.132
Age: 45-54	0.446	[0.277-0.719]	0.001**	1.266	[0.803-1.998]	0.310
Age: 55-64	0.181	[0.102-0.323]	0.000***	1.152	[0.647-2.052]	0.630
Age: 65+	0.095	[0.027-0.340]	0.000***	0.948	[0.430-2.089]	0.894
Women	0.992	[0.766-1.283]	0.949	1.075	[0.850-1.359]	0.547
Experience of adverse effects				1.009	[0.774-1.315]	0.946

***p < 0.001, **p < 0.01, *p < 0.05.

Results are based on two-tailed tests. Cox proportional hazard regressions were conducted using R.

Table S4. Structural equation model predicting vaccination delay (with post-mandate data)

	First dose (N = 286)[a] CFI = .964, TLI = .951, RMSEA = .044, SRMR = .050				Third dose (N = 341)[b] CFI = .935, TLI = .911, RMSEA = .053, SRMR = .061			
	Estimate†	SE	z	p	Estimate†	SE	z	p
Vaccination delay ~								
Intention	-0.078	0.003	-2.254	0.024*	-0.173	0.003	-2.515	0.012*
Trust	-0.047	0.011	-1.453	0.146	-0.098	0.012	-1.498	0.134
Confidence	0.013	0.008	0.283	0.777	-0.024	0.010	-0.273	0.785
Complacency	-0.019	0.011	-0.407	0.684	0.103	0.011	1.138	0.255
Collective responsibility	-0.060	0.011	-1.332	0.183	0.331	0.015	3.236	0.001**
Calculation	-0.008	0.010	-0.254	0.800	-0.185	0.012	-2.586	0.010*
Constraint	0.059	0.009	1.450	0.147	-0.023	0.010	-0.269	0.788
Cluster	0.823	0.024	23.625	0.000***				
Age (25-34)	0.124	0.022	3.365	0.001**	-0.041	0.027	-0.446	0.656
Age (35-44)	0.058	0.023	1.625	0.104	-0.120	0.028	-1.367	0.172
Age (45-54)	0.079	0.030	2.522	0.012*	-0.110	0.032	-1.310	0.190
Age (55-64)	0.100	0.034	3.125	0.002**	-0.034	0.041	-0.514	0.607
Age (65-74)	0.029	0.077	1.051	0.293	-0.030	0.054	-0.499	0.618
Women	0.024	0.017	0.850	0.395	-0.052	0.016	-0.958	0.338
Round	0.029	0.011	0.768	0.443	-0.010	0.018	-0.174	0.862
Experience of adverse effects					0.001	0.018	0.022	0.982
Intention ~								
Confidence	0.532	0.147	6.761	0.000***	0.179	0.195	2.425	0.015*
Complacency	-0.293	0.206	-3.455	0.001**	-0.017	0.203	-0.223	0.823
Collective responsibility	-0.085	0.223	-0.985	0.324	0.269	0.268	3.253	0.001**
Calculation	-0.025	0.203	-0.422	0.673	-0.058	0.228	-0.990	0.322
Constraint	0.092	0.169	1.196	0.232	-0.108	0.192	-1.545	0.122
Trust	0.042	0.219	0.685	0.494	0.267	0.209	4.949	0.000***
Age (25-34)	-0.095	0.415	-1.377	0.168	0.067	0.501	0.886	0.376
Age (35-44)	-0.097	0.444	-1.453	0.146	0.158	0.512	2.192	0.028*
Age (45-54)	-0.017	0.579	-0.292	0.770	0.240	0.589	3.502	0.000***
Age (55-64)	0.033	0.647	0.557	0.578	0.055	0.750	1.000	0.317
Age (65-74)	0.022	1.483	0.431	0.666	0.088	0.992	1.763	0.078
Women	0.052	0.327	0.957	0.339	0.004	0.304	0.087	0.931
Round	-0.004	0.176	-0.062	0.950	0.159	0.325	3.372	0.001**
Experience of adverse effects					-0.046	0.335	-1.006	0.315
Confidence ~								

Table S4. Structural equation model predicting vaccination delay (with post-mandate data)

Trust	0.381	0.121	5.988	0.000***	0.331	0.091	5.326	0.000***
Age (25-34)	-0.023	0.253	-0.292	0.770	-0.175	0.231	-1.888	0.059
Age (35-44)	-0.031	0.272	-0.402	0.687	-0.095	0.238	-1.068	0.286
Age (45-54)	0.053	0.351	0.796	0.426	-0.233	0.263	-2.875	0.004**
Age (55-64)	-0.065	0.384	-0.986	0.324	0.009	0.345	0.129	0.897
Age (65-74)	0.074	0.899	1.255	0.210	0.008	0.462	0.126	0.900
Women	-0.268	0.187	-4.635	0.000***	-0.141	0.139	-2.560	0.010*
Round	-0.097	0.092	-1.699	0.089	-0.073	0.147	-1.301	0.193
Experience of adverse effects					0.004	0.156	0.065	0.948
Complacency ~								
Trust	0.064	0.095	0.984	0.325	0.006	0.094	0.083	0.934
Age (25-34)	0.072	0.212	0.844	0.399	0.172	0.252	1.650	0.099
Age (35-44)	0.064	0.228	0.764	0.445	0.075	0.259	0.744	0.457
Age (45-54)	0.109	0.296	1.488	0.137	0.127	0.286	1.400	0.161
Age (55-64)	0.114	0.324	1.587	0.112	0.033	0.376	0.434	0.664
Age (65-74)	-0.043	0.756	-0.667	0.505	-0.039	0.504	-0.560	0.575
Women	0.085	0.156	1.355	0.175	0.053	0.150	0.862	0.389
Round	0.266	0.079	4.170	0.000***	0.246	0.168	3.691	0.000***
Experience of adverse effects					0.010	0.170	0.151	0.880
Collective responsibility ~								
Trust	0.130	0.081	2.202	0.028*	0.192	0.079	2.911	0.004**
Age (25-34)	-0.069	0.181	-0.889	0.374	-0.027	0.205	-0.271	0.786
Age (35-44)	-0.062	0.193	-0.825	0.409	-0.022	0.213	-0.231	0.818
Age (45-54)	-0.042	0.251	-0.629	0.529	0.020	0.234	0.224	0.823
Age (55-64)	-0.069	0.273	-1.074	0.283	0.010	0.309	0.137	0.891
Age (65-74)	0.033	0.643	0.560	0.575	0.021	0.413	0.307	0.759
Women	-0.031	0.132	-0.545	0.586	-0.022	0.124	-0.363	0.717
After vaccine mandates	-0.457	0.074	-7.187	0.000***	-0.073	0.130	-1.189	0.234
Experience of adverse effects					0.003	0.139	0.049	0.961
Calculation ~								
Trust	-0.206	0.068	-3.134	0.002**	-0.096	0.063	-1.517	0.129
Age (25-34)	0.039	0.149	0.456	0.649	0.075	0.167	0.763	0.445
Age (35-44)	0.055	0.160	0.663	0.507	0.016	0.173	0.172	0.864
Age (45-54)	0.063	0.207	0.861	0.389	0.016	0.191	0.182	0.856
Age (55-64)	0.052	0.225	0.731	0.465	0.116	0.252	1.603	0.109
Age (65-74)	0.072	0.531	1.119	0.263	0.033	0.336	0.510	0.610
Women	0.060	0.109	0.964	0.335	-0.028	0.101	-0.481	0.631
After vaccine mandates	-0.095	0.054	-1.523	0.128	0.040	0.106	0.679	0.497
Experience of adverse effects					0.077	0.113	1.299	0.194

Table S4. Structural equation model predicting vaccination delay (with post-mandate data)

Constraint ~								
Trust	0.253	0.104	3.937	0.000***	-0.009	0.092	-0.132	0.895
Age (25-34)	0.127	0.225	1.538	0.124	0.078	0.243	0.776	0.438
Age (35-44)	0.079	0.242	0.991	0.322	0.035	0.251	0.358	0.721
Age (45-54)	0.005	0.313	0.077	0.938	-0.029	0.277	-0.330	0.742
Age (55-64)	-0.130	0.342	-1.885	0.059	-0.112	0.365	-1.520	0.128
Age (65-74)	-0.127	0.803	-2.060	0.039*	-0.062	0.489	-0.915	0.360
Women	-0.009	0.165	-0.158	0.874	0.048	0.146	0.809	0.419
After vaccine mandates	0.204	0.082	3.396	0.001**	0.149	0.156	2.395	0.017*
Experience of adverse effects					0.047	0.164	0.767	0.443

***p < 0.001, **p < 0.01, *p < 0.05. [†]Standardized estimates with respect to the observed and latent variables

[a] Because of the bimodal nature of the outcome variable, mixture modelling was used.

[b] Because of the Poisson nature of the outcome variable, the outcome variable was sqrt-transformed.

Results are based on two-tailed tests. The structural equation models were conducted using R.

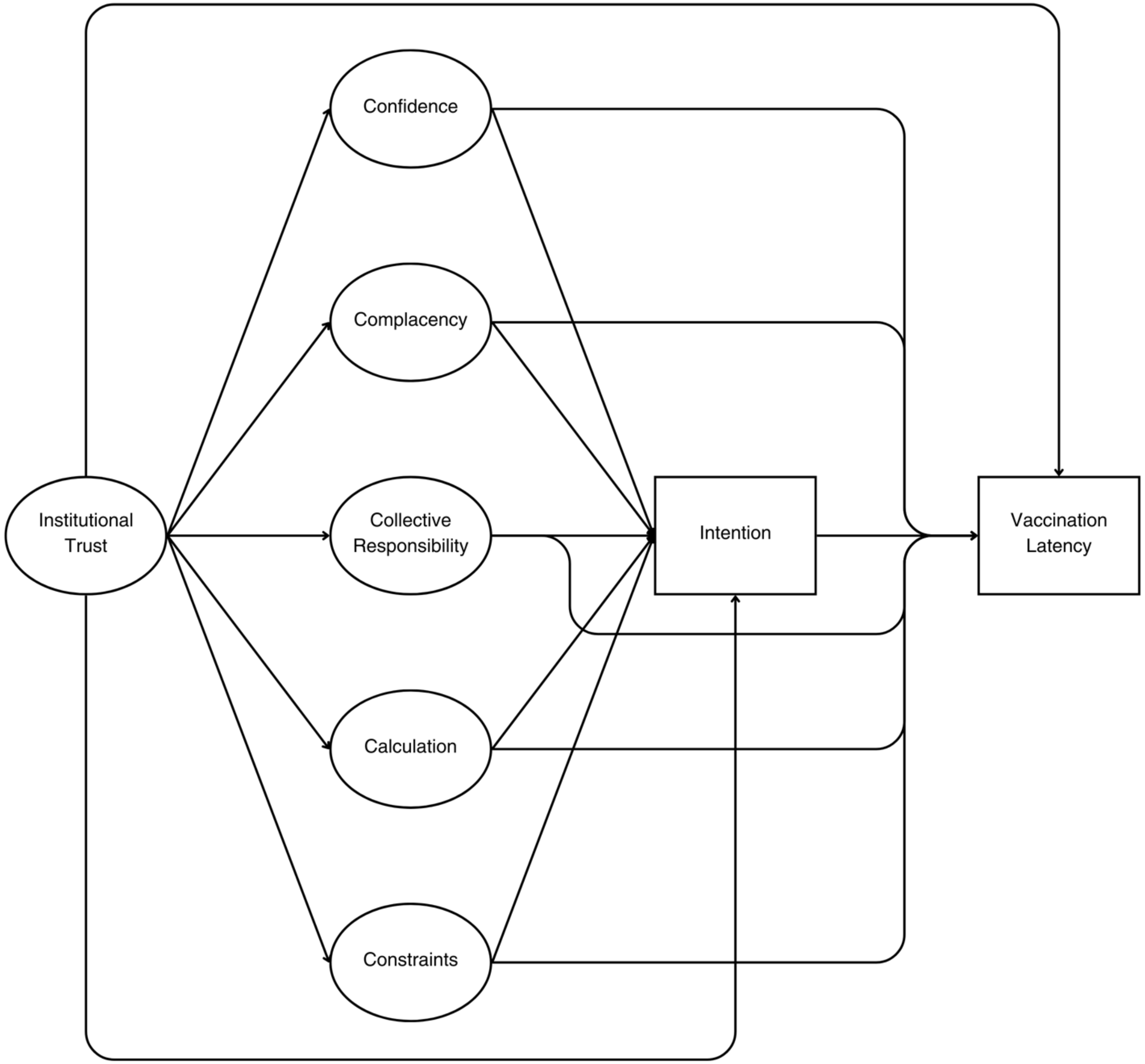

Figure S1. Graphical illustration of the structural equation model examined

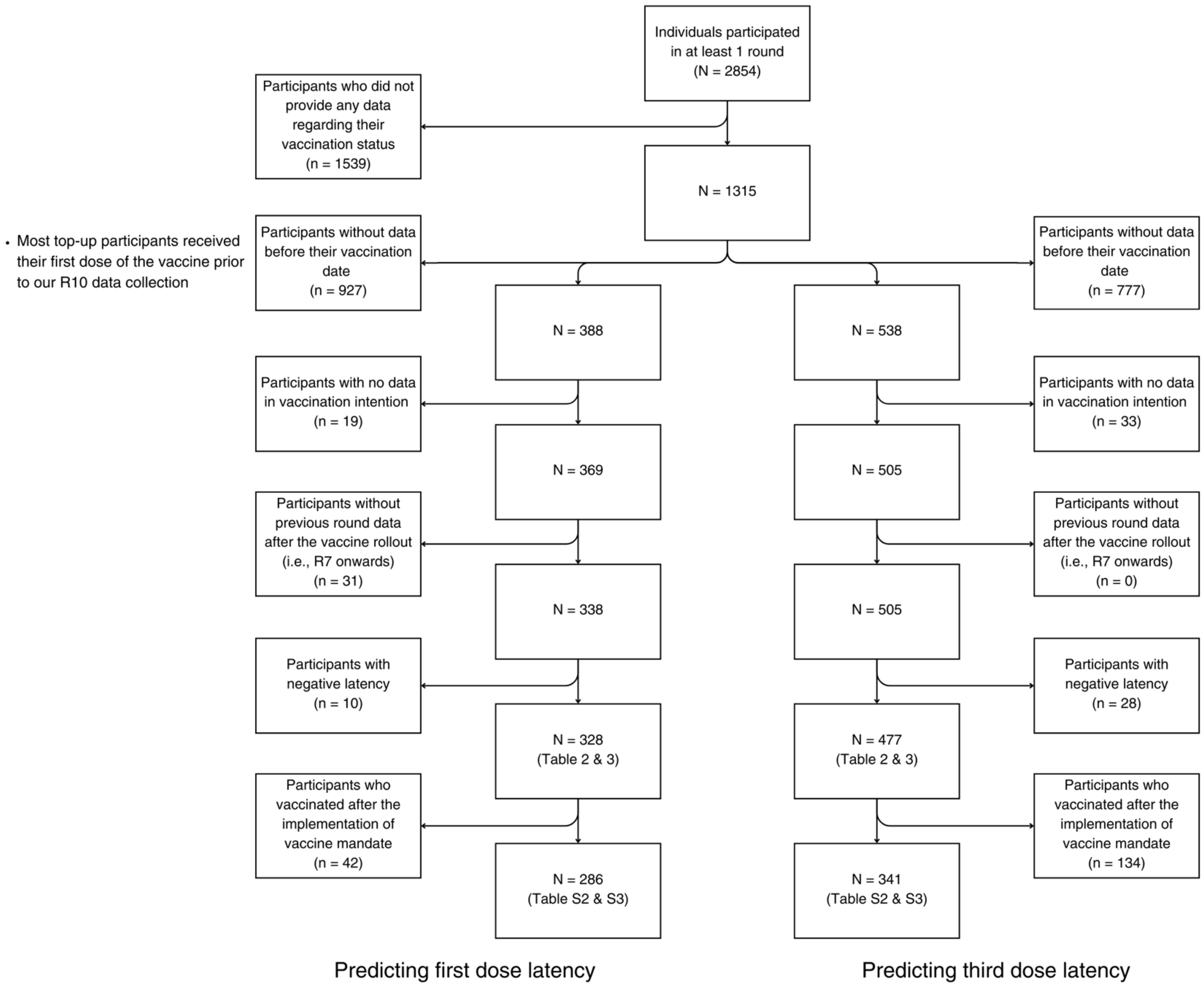

Figure S2. Sample size flow chart

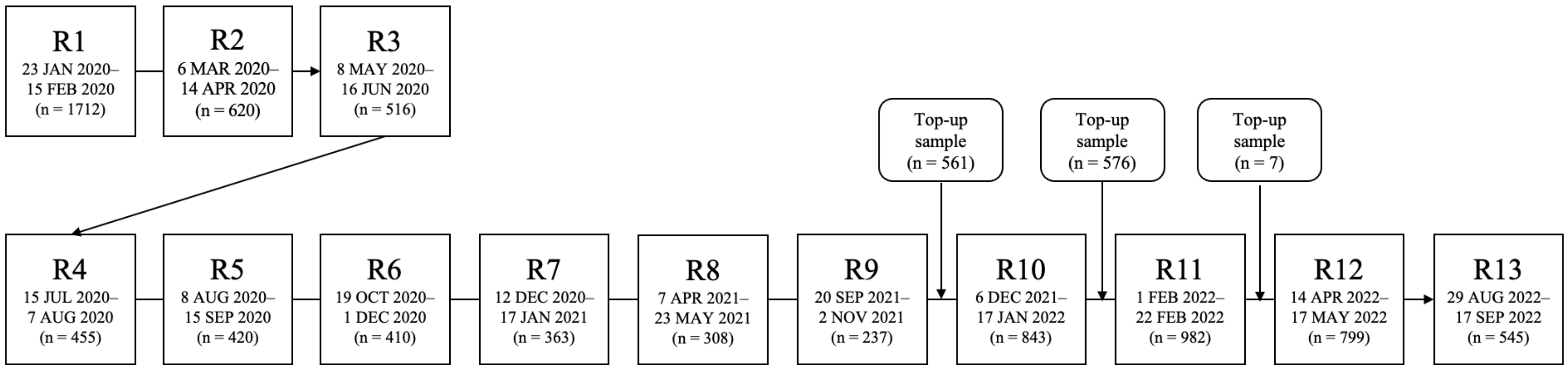

Figure S3. Data collection of the 13 rounds of the community survey.

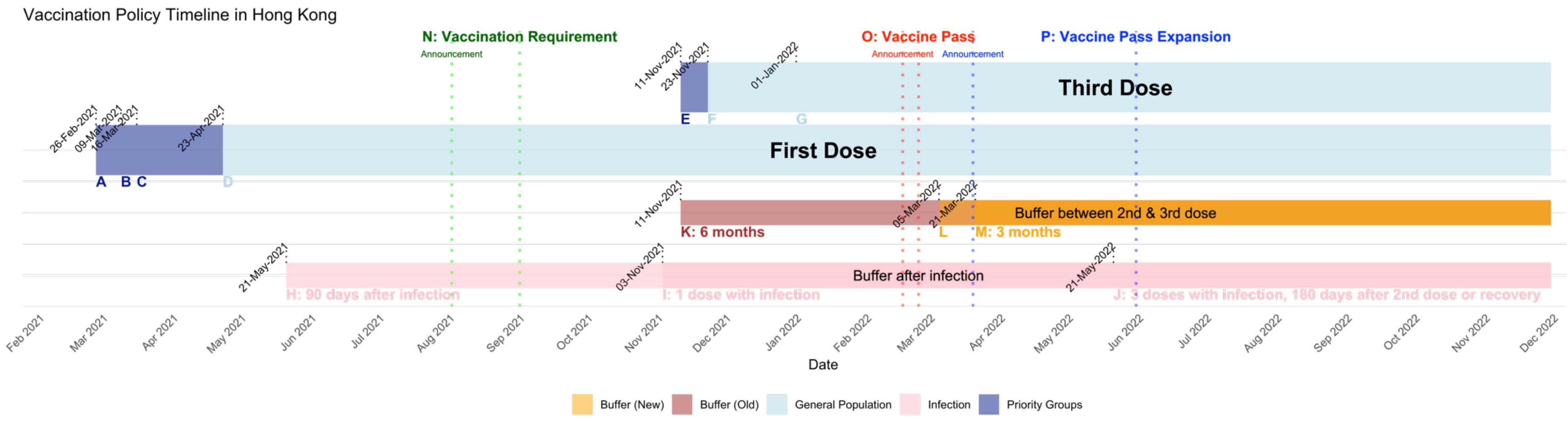

Figure S4. Vaccination policy timeline in Hong Kong

Notes.

<u>*First Dose*</u>

A. From 26 February 2021 onwards, five priority groups can receive the Sinovac vaccine [16]:

1) Healthcare staff and staff involved in anti-epidemic work;
2) Persons aged 60 or above (A maximum of two carers who accompany elderly people aged 70 or above can also receive vaccination);
3) Residents and staff of residential care homes for the elderly and persons with disabilities;
4) People providing essential public services;
5) People providing cross-boundary transportation or working at control points and ports.

B. From 9 March 2021 onwards, these seven categories of priority groups can make reservations through the online booking system for receiving vaccines at community vaccination centres or they can choose to get vaccinated at clinics of private doctors[24]:

1) Staff of food and beverages premises, markets, supermarkets, convenience stores, couriers and takeaway delivery (including takeaway food delivery);
2) Staff of local public transport service operators (e.g. taxi/bus/public light bus drivers, train captains and station staff);
3) Registered construction workers;
4) Staff of property management (e.g. security guards and cleaning and security staff);
5) Teachers and school staff (e.g. teaching and support staff of kindergartens, primary and secondary schools and universities; staff of special schools; and drivers and escorts of school buses and school private light buses);
6) Staff in the tourism industry; and
7) Staff of scheduled premises under the Prevention and Control of Disease (Requirements and Directions) (Business and Premises) Regulation (Cap. 599F) (e.g. staff of fitness centres and beauty parlours).

C. From 16 March 2021 onwards, the priority groups expanded to cover the following categories [25]:

1) Persons aged 30 years or above (a maximum of two carers who accompany elderly people aged 70 or above can also receive vaccination);
2) Personnel in healthcare settings and those participating in anti-epidemic related work;
3) Residents and staff of residential care homes for the elderly/residential care homes for persons with disabilities and staff of community care services units for the elderly/persons with disabilities;

4) Personnel maintaining critical public services;
5) Personnel providing cross-boundary transportation or working at control points and ports;
6) Staff of food and beverages premises, markets, supermarkets, convenience stores and couriers (including takeaway food delivery);
7) Staff of local public transport service operators;
8) Registered construction workers and other resident site personnel;
9) Staff of property management (including security and cleaning staff);
10) Teachers and school staff;
11) Staff of the tourism industry;
12) Staff of scheduled premises under the Prevention and Control of Disease (Requirements and Directions) (Business and Premises) Regulation (Cap. 599F) (including bathhouses, indoor places of public entertainment, clubs/nightclubs, karaoke establishments and swimming pools) ;
13) Students studying outside Hong Kong (aged 16 or above); and
14) Domestic helpers.

D. From 23 April 2021 onwards, the COVID-19 Vaccination Programme expanded to cover persons aged 16 to 29 [26]. The minimum age for receiving the BioNTech vaccine is 16, and that for receiving the Sinovac vaccine is 18.

Third Dose

E. From 11 November 2021 onwards, a third dose of a COVID-19 vaccine are available for individuals in the following priority groups [11]:

1) Certain groups of immunocompromised patients (e.g. cancer patients, organ transplant recipients, advanced-stage HIV patients and patients taking active immunosuppressive drugs). The third dose should be administered at least four weeks from the second dose.
2) Those who have received two doses of Sinovac vaccine and with a higher risk of infection, including elderly aged 60 or above; healthcare workers; persons with chronic illnesses; and workers at increased risk for COVID-19 exposure and transmission because of occupational setting (e.g. personnel participating in anti-epidemic related work; personnel providing cross-boundary transportation or working at control points and ports). The third dose should be administered at least six months after the second dose.

F. From 23 November 2021 onwards, members of the public who have received two doses of the Sinovac vaccine with the second dose received six months ago, irrespective of whether they belong to the certain groups, can make reservations for and receive a third dose of a COVID-19 vaccine [27].

G. From 1 January 2022 onwards, Provision of a third dose vaccination service to all eligible persons who have received two doses of the BioNTech vaccine with the second dose received six months ago [28].

Buffer after Infection

H. As announceed on 21 May 2021, recovered persons who wish to receive the BioNTech vaccine should wait for at least 90 days after discharge from previous infection [29].

I. As announced on 3 November 2021, individuals who are fully recovered from COVID-19 are only required to receive one dose of vaccine. Recovered patients should discuss with their physician whether two doses of COVID-19 vaccine are required, and an interval of six months between the two doses is recommended [11].

J. As announced on 21 May 2022, for recovered adults in the same age group (i.e. aged 18 to 59), they can receive their third dose six months after they have received their second dose or their recovery (whichever is later) [30].

Buffer after the 2nd Dose

K. As from 11 November 2021, certain groups of immunocompromised patients as indicated in POLICY E can adminstered the third dose at least four weeks from the second dose. For individuals aged 18 or above in the aforementioned higher risk populations in POLICY E who have received two doses of Fosun/BioNTech vaccine, an additional dose of Fosun/BioNTech vaccine administered at least six months from the second dose is recommended [11]. These persons may, however, choose Sinovac vaccine as the third dose.

L. As from 5 March 2022, the intervals between COVID-19 vaccine doses are shortened [31]:

1) For persons aged 60 or above who have received two doses of the Sinovac or the BioNTech vaccine, the time interval for receiving the third dose will be shortened from six months after the second dose to three months;

2) Immunocompromised children aged below 12 can make appointments for receiving the third dose four weeks after receiving the first two doses of the Sinovac or the BioNTech vaccine.

M. As from 21 March 2022, persons aged 18 to 59 who have already received two doses of a COVID-19 vaccine can make appointments for the third dose three months after their second dose [32].

Vaccination Requirements

N. The HKSAR Government announced the vaccination requirement for all civil servants on **2 August 2021** and in effect on **1 September 2021** [13]. Major corporations in Hong Kong have followed and implemented the same requirement to their employees on the same date.

O. The implementation of the vaccine pass (for the first two doses of the COVID-19 vaccines) was announced on **17 February 2022** and in effect on **24 February 2022** [14].

P. The adjustment of the vaccine pass that included the third dose in the mandate was announced on **20 March 2022** and in effect on **31 May 2022** [15].

Buffer Period After Infection

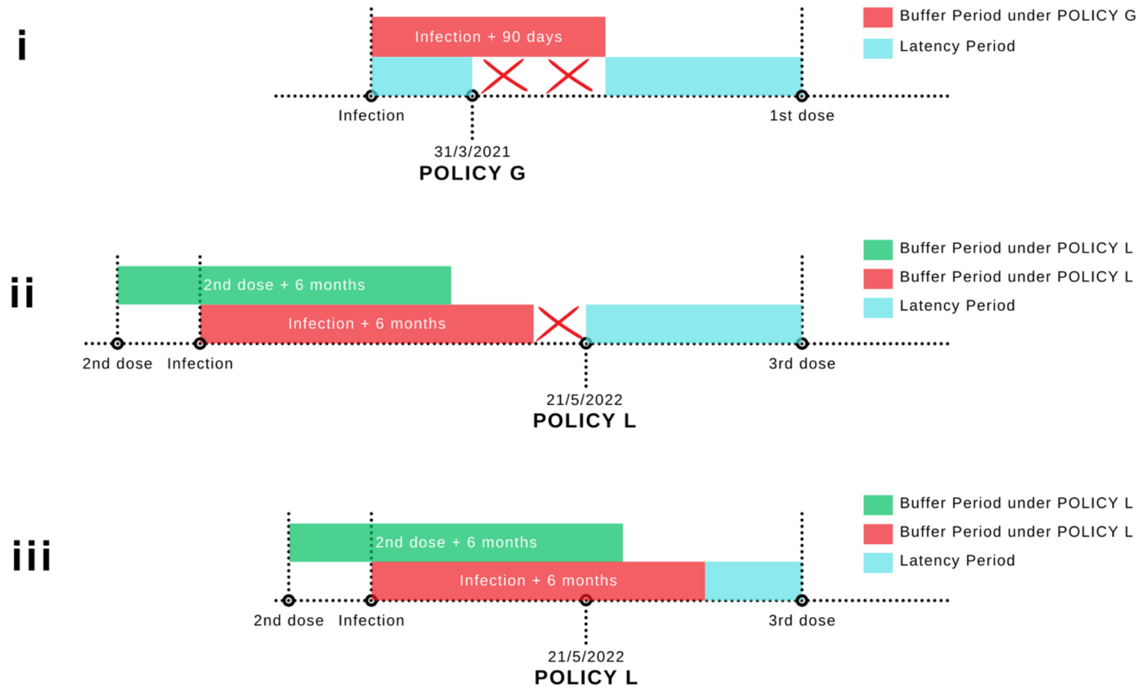

Figure S5i. Scenarios for calculating vaccination delay

Notes.

Buffer after infection

i. The infection happened before POLICY G was implemented: The individual was suggested to take the 1st dose immediately after the infection + 90 days buffer period. The gap between the infection and the implementation of POLICY G is also counted as the vaccination delay period.

ii. The infection happened more than 6 months before POLICY L was implemented (for an individuals vaccinated with the 2nd dose): The individual was eligible to take the 3rd dose immediately on 21/5/2022.

iii. The infection happened less than 6 months before POLICY L was implemented (for an individuals vaccinated with the 2nd dose): The individual was eligible to take the 3rd dose after the infection + 6 months buffer period.

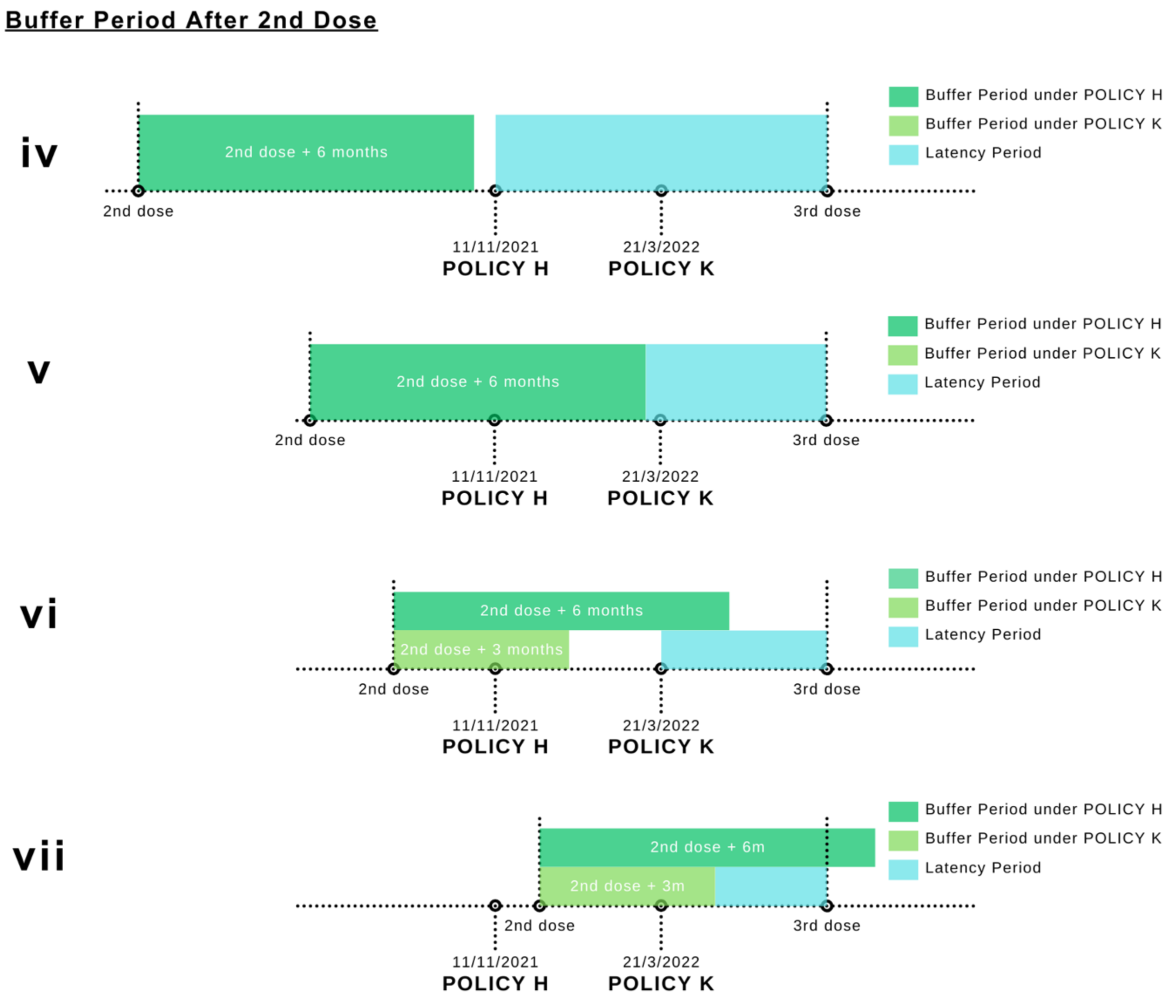

Figure S5ii. Scenarios for calculating vaccination delay

Notes.

Buffer after 2nd dose

iv. The 2nd dose was taken more than 6 months before POLICY H was implemented: The individual was eligible to take the 3rd dose immediately on 11/11/2021.

v. The 2nd dose was taken less than 6 months before POLICY H was implemented (but more than 6 months before POLICY K was implemented): The individual was eligible to take the 3rd dose after the 2nd dose + 6 month buffer period.

vi. The 2nd dose was taken less than 6 months (but more than 3 months) before POLICY K was implemented: The individual was eligible to take the 3rd dose immediately on 21/3/2022.

vii. The 2nd dose was taken less than 3 months before POLICY K was implemented: The individual was eligible to take the 3rd dose after the 2nd dose + 3 months buffer period.

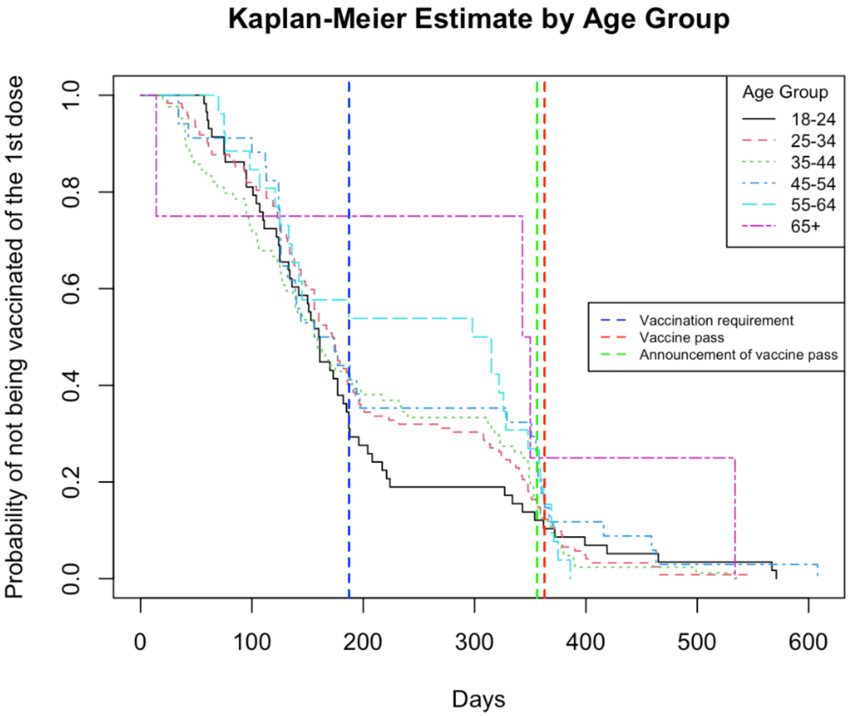

First Dose

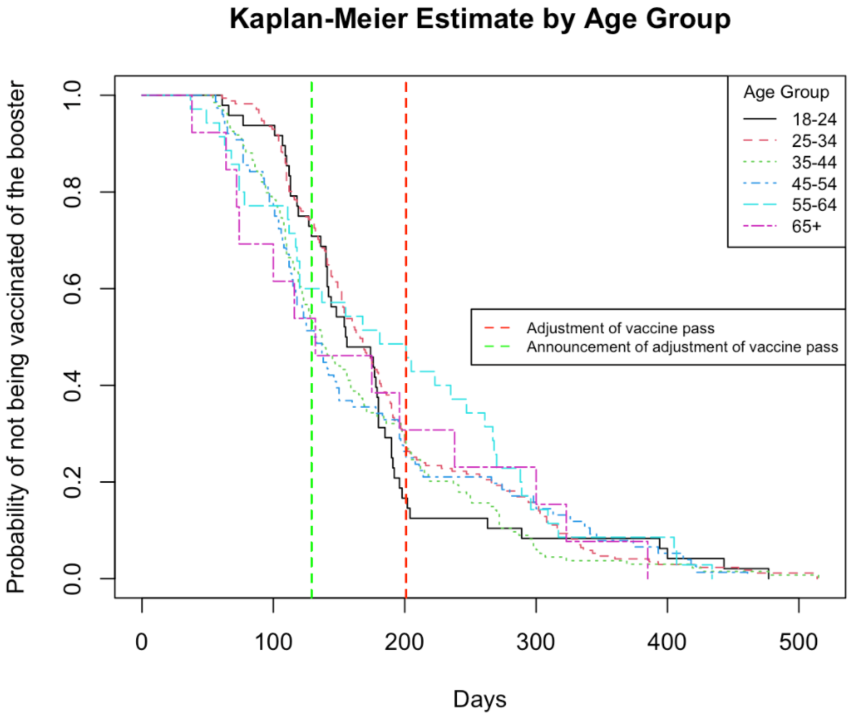

Third Dose

Figure S6. Kaplan-Meier survival curves of unadjusted vaccination delay by age groups (i.e., days lapsed since the very first available dates of the first dose and booster in Hong Kong)
